# Aerobic exercise, whey protein, and creatine in adolescents: implications for performance, recovery, immunometabolic adaptation, and muscle–bone–brain crosstalk

**DOI:** 10.3389/fnut.2026.1915252

**Published:** 2026-09-25

**Authors:** Kun Qin, Juan Wu

**Affiliations:** College of Physical Education, Beihua University, Jilin City, Jilin, China

**Keywords:** adolescents, aerobic exercise, creatine, energy dynamics, metabolism, movement, whey protein

## Abstract

Adolescence constitutes a pivotal developmental phase for the enhancement of musculoskeletal and metabolic wellbeing, as well as neurodevelopment, during which specific exercise regimens and nutritional interventions can precipitate enduring physiological transformations. Although aerobic exercise has conventionally been linked to enhancements in cardiovascular fitness and metabolic control, the confluence of this form of exercise with anabolic and ergogenic supplements, including whey protein and creatine, in adolescent populations has not been extensively investigated. This narrative review examines current evidence on aerobic exercise, whey protein, and creatine in adolescents, with particular emphasis on exercise performance, recovery, immunometabolic adaptation, and muscle–bone–brain crosstalk. A narrative literature search was conducted in PubMed, Scopus, Web of Science, and Google Scholar using combinations of terms related to aerobic exercise, whey protein, creatine, adolescents, exercise performance, recovery, immunometabolism, and muscle–bone–brain signaling. Relevant peer-reviewed studies published from database inception to May 2026 were considered, with emphasis on adolescent human studies and selected mechanistic evidence from experimental and adult populations when directly relevant to biological interpretation. Aerobic exercise promotes oxidative metabolic processes, mitochondrial functionality, and endurance performance, thereby enhancing mitochondrial biogenesis and metabolic flexibility. Whey protein supplies critical amino acids that incite muscle protein turnover and modulate pivotal signaling cascades, thereby supporting muscle remodeling and recovery. Creatine, in addition to its function in the swift regeneration of adenosine triphosphate, plays a significant role in cellular hydration, the preservation of mitochondrial integrity, and the maintenance of antioxidant defense mechanisms, while also potentially influencing brain energy metabolism and brain-derived neurotrophic factor-mediated neuroplasticity. Emerging evidence further suggests that exercise and nutritional interventions may influence inflammatory mediators, myokines, osteokines, and neurotrophic pathways involved in muscle–bone–brain communication. However, mechanistic evidence is frequently derived from animal or non-adolescent human studies, and direct evidence for combined interventions in adolescents remains limited. Overall, aerobic exercise, whey protein, and creatine may have complementary roles in supporting exercise performance, recovery, and metabolic adaptation during adolescence, although further well-controlled adolescent studies are needed to establish their long-term efficacy, safety, and optimal use.

## 1 Introduction

Adolescence constitutes a pivotal developmental phase distinguished by accelerated growth, hormonal variations, and profound physiological modifications that collectively influence metabolic processes, musculoskeletal maturation, neuromotor coordination, and brain development. During this critical period, engagement in organized physical activity, particularly aerobic exercise, plays a fundamental role in shaping health trajectories, athletic performance, and long-term functional capacity. Nevertheless, the escalating demands imposed on adolescent athletes, including intensified training schedules, competitive pressures, and early sport specialization, have heightened interest in nutritional strategies capable of supporting optimal growth, recovery, and performance outcomes. Among these interventions, dietary supplementation with whey protein and creatine has attracted substantial attention owing to their established roles in energy metabolism, muscle protein synthesis (MPS), and functional performance enhancement (1, 2). The rationale for nutritional supplementation among adolescent athletes is related to the distinctive physiological and metabolic requirements of this developmental period. Adolescents frequently have elevated energy needs to support both growth and physical activity; however, dietary intake may be inadequate or unbalanced because of lifestyle factors, limited nutritional knowledge, or restrictive eating habits (3, 4). Dietary protein is particularly important during adolescence because it supports muscle growth, tissue remodeling, and exercise recovery. Whey protein, characterized by high biological value and rapid digestibility, is rich in essential amino acids, particularly leucine, and can stimulate MPS, primarily through activation of the mechanistic target of rapamycin (mTOR) signaling pathway (5, 6). Likewise, creatine, a naturally occurring compound involved in phosphocreatine energy metabolism, facilitates rapid adenosine triphosphate (ATP) regeneration during high-intensity physical activity and has been associated with improvements in strength, power, and lean body mass (7, 8).

Emerging research indicates that, although these supplements have been studied predominantly in adults, they may also provide benefits for selected adolescent athletes when used appropriately and under suitable supervision (9). The interaction between physical activity and dietary intake is particularly relevant during adolescence because of the substantial physiological adaptability that characterizes this developmental stage. Aerobic exercise promotes several metabolic adaptations, including enhanced mitochondrial biogenesis, improved oxidative capacity, and more efficient energy utilization (10, 11). Nutritional factors may further modulate these adaptations; for example, adequate protein availability after aerobic or mixed-modal exercise may support muscle recovery and remodeling, whereas creatine supplementation may contribute to neuromuscular performance and cellular bioenergetics (12, 13). These responses involve signaling pathways such as AMP-activated protein kinase (AMPK) and peroxisome proliferator-activated receptor gamma coactivator-1 alpha (PGC-1α), which are important regulators of cellular energy sensing and mitochondrial adaptation (14–16). Myokines and other exercise-responsive mediators, including irisin, interleukin-6 (IL-6), and brain-derived neurotrophic factor (BDNF), have also been proposed as links between physical activity, metabolism, musculoskeletal function, and brain adaptation (17, 18).

The muscle–bone–brain axis has emerged as a useful conceptual framework for understanding how exercise-related signals may connect skeletal muscle, bone, and the nervous system. Through interactions involving myokines, osteokines, neurotrophins, and inflammatory mediators, exercise and nutritional status may influence musculoskeletal integrity, metabolic regulation, and neuroplasticity (19). However, the strength of evidence supporting these connections differs substantially across biological models and populations. Some pathway-level evidence comes from animal experiments or adult human studies rather than adolescents, and these findings should therefore be interpreted as mechanistic evidence rather than direct demonstrations of adolescent responses. Skeletal muscle functions as an endocrine organ and releases bioactive molecules in response to contraction that can affect distant tissues, including the brain, liver, and adipose tissue (17). These signals may contribute to the regulation of energy homeostasis, inflammation, and neuroplasticity. Nutritional availability may additionally shape these inter-organ responses by influencing substrate supply and cellular energy metabolism (20).

Motor competence is another potentially relevant outcome of exercise and nutritional status during adolescence. However, the available adolescent literature does not consistently include direct assessments of validated constructs such as physical literacy, motor learning, or coordination. Instead, studies have more commonly examined performance-related or motor competence-related measures, including sprint performance, jumping ability, dribbling, repeated-sprint performance, aerobic capacity, and attention (21, 22). Accordingly, these outcomes are considered in this review primarily as indicators of functional and sport-related performance rather than as direct measures of physical literacy. This distinction is important when interpreting evidence linking aerobic exercise, whey protein, and creatine with broader developmental outcomes.

Although interest in exercise nutrition during adolescence has increased, previous reviews have generally focused on individual supplements, isolated exercise outcomes, or predominantly adult populations. In contrast, the present narrative review brings together adolescent evidence on three complementary components (aerobic exercise, whey protein, and creatine) while examining their potential relationships with performance and recovery, immunometabolic adaptation, and muscle–bone–brain communication. A further distinctive feature of this review is its explicit separation of adolescent human evidence from mechanistic findings derived from animal or non-adolescent populations. This approach is intended to distinguish established adolescent findings from biologically plausible mechanisms that remain to be directly demonstrated in adolescents.

The review also considers the potential relevance of mTOR-, AMPK-, and PGC-1α-related signaling, inflammatory mediators, myokines, osteokines, and neurotrophic factors as mechanistic links between exercise, nutritional status, and physiological adaptation. Rather than assuming that aerobic exercise, whey protein, and creatine produce synergistic effects, we evaluate whether the available evidence supports complementary or potentially interacting roles of these interventions. Furthermore, safety, appropriate use, dosage considerations, and the limited evidence on long-term developmental consequences are considered because adolescents represent a still-developing population (23).

Therefore, the purpose of this narrative review is to critically synthesize current evidence on aerobic exercise, whey protein, and creatine in adolescents, with particular emphasis on exercise performance, recovery, immunometabolic adaptation, and muscle–bone–brain crosstalk. By integrating adolescent clinical and physiological evidence with selected mechanistic studies, while clearly distinguishing direct evidence from extrapolated biological mechanisms, this review aims to provide a balanced framework for understanding the potential roles and limitations of exercise and nutritional strategies during adolescence.

## 2 Literature search strategy

This article was conducted as a narrative review to synthesize current evidence on aerobic exercise, whey protein, and creatine in adolescents, with particular emphasis on performance, recovery, immunometabolic adaptation, and muscle–bone–brain crosstalk. Because the objective was to provide a critical and integrative synthesis rather than a systematic quantitative assessment, the review did not follow a formal systematic-review protocol or PRISMA framework.

A structured literature search was conducted using PubMed/MEDLINE, Scopus, Web of Science, and Google Scholar. The search covered publications available from database inception to May 2026. Search terms were developed around four major concepts: (1) aerobic exercise and physical activity, (2) whey protein and dietary protein, (3) creatine and creatine supplementation, and (4) adolescence and adolescent athletes. Additional terms related to the major outcomes and mechanistic themes of the review were also considered, including performance, recovery, muscle strength, aerobic capacity, immunometabolism, inflammation, AMPK, mTOR, PGC-1α, myokines, osteokines, BDNF, neuroplasticity, muscle–bone–brain axis, *and* motor competence. Boolean operators (AND/OR) were used to combine the major concepts and broaden or refine the search where appropriate.

The literature searches and subsequent screening focused primarily on human studies involving children, adolescents, or adolescent athletes, particularly studies evaluating aerobic or endurance exercise, whey protein, creatine supplementation, or combinations of exercise and nutritional interventions. Studies in adults and older populations were considered when they provided important mechanistic or biological evidence relevant to pathways discussed in the review, including mTOR–AMPK–PGC-1α signaling, myokine and osteokine signaling, neurotrophic mechanisms, and muscle–bone–brain communication. Because such evidence was not directly derived from adolescents, these studies were used to establish biological plausibility and were explicitly distinguished from adolescent-specific evidence in the manuscript.

Studies were selected when they were relevant to the central objectives of the review and provided information on exercise performance, recovery, body composition, metabolic or inflammatory responses, bone-related outcomes, neuromuscular function, or mechanistic pathways relevant to the proposed framework. Peer-reviewed original research articles, randomized or controlled trials, observational studies, and relevant mechanistic studies were considered. Relevant review articles and position statements were also consulted to provide background context and to identify additional primary studies.

Studies were excluded when they were unrelated to the central population or interventions, focused exclusively on non-exercise-related supplementation, lacked relevance to the outcomes or mechanisms addressed in the review, or were non-peer-reviewed sources without sufficient scientific information. When evidence from non-adolescent populations or animal models was incorporated, it was used selectively for mechanistic interpretation rather than as direct evidence of efficacy or safety in adolescents.

The final body of literature was critically synthesized according to the major themes of the review. Evidence summarized in Tables 1–8 was selected based on its relevance to adolescent supplementation and exercise responses, immunometabolic mediators, muscle–bone–brain signaling, and motor competence-related outcomes. Given the narrative nature of this review, no formal meta-analysis was performed, and the available evidence was interpreted qualitatively with particular attention to population characteristics, experimental models, intervention protocols, outcome measures, and the distinction between direct adolescent evidence and extrapolated mechanistic findings.

**TABLE 1:** Effects of whey protein supplementation and exercise in adolescents.

Population	Intervention	Study design	Key outcomes measured	Main results	Conclusion	Study
*n* = 5; late adolescents/young adults with Barth syndrome (27 ± 7 yrs)	12 weeks resistance exercise training (RET) + 42 g/day whey protein	Pre–post pilot study	Muscle strength, lean mass, exercise capacity, cardiac function, QOL	↑ Muscle strength; ↑ QOL; trend toward ↑ lean mass; safe intervention	RET + protein is safe and improves strength and QOL; larger studies needed	(59)
*n* = 54 swimmers (11–17 yrs)	Post-exercise whey protein vs. carbohydrate vs. water	Randomized controlled trial	Performance (200 m), CK, IL-6, IL-10, TNF-α, muscle soreness	No performance differences; ↓ muscle soreness (PRO vs. H_2_O); ↑ IL-10 (anti-inflammatory)	No major recovery benefit vs. carbs, but may support anti-inflammatory response	(57)
*n* = 58 swimmers (14.1 ± 0.4 yrs)	Post-exercise whey protein vs. carbohydrate vs. water	Controlled trial	PINP, CTXI, bone turnover rate	No change in PINP; ↓ CTXI at 24 h (protein only); ↑ bone turnover rate	Protein may promote positive bone turnover after intense exercise	(55)
*n* = 22 soccer players (15.6 ± 0.2 yrs; 59% female)	Whey protein (20 g, twice daily) vs. isocaloric placebo for 10 weeks	Randomized controlled trial	V̇O_2_max, sprint time, muscle strength, endurance, body composition	↑ V̇O_2_max (greater in PRO); no difference in sprint or strength; ↓ muscular endurance in PRO; ↑ fat-free mass (both groups)	Whey protein improved aerobic capacity but not strength, sprint, or body composition vs. control	(58)
*n* = 47 athletes (∼16 yrs; both sexes)	Greek yogurt (2 × 175 g/day) vs. isonitrogenous whey protein for 16 weeks	Randomized intervention (after control period)	Bone markers (CTX-I, osteocalcin, OPG), inflammatory markers (IL-1β, IL-6, IL-10, TNF-α), body composition	Modest, sex-specific changes in bone markers; CTX-I transient ↑ in GY; inflammatory markers varied (↓ IL-6 in GY, ↑ IL-1β in WP)	Protein intake (regardless of source) induces modest, variable effects on bone and inflammation	(56)

RET, resistance exercise training; PRO, protein group; CHO, carbohydrate group; H_2_O, water/placebo; GY, Greek yogurt; WP, whey protein; QOL, quality of life; V̇O_2_max, maximal oxygen uptake; CK, creatine kinase; IL, interleukin; TNF-α, tumor necrosis factor-alpha; PINP, procollagen type I N-terminal propeptide; CTX-I, C-terminal telopeptide of type I collagen; OPG, osteoprotegerin.

**TABLE 2:** Methodological characteristics, risk of bias, and overall quality of included studies evaluating protein-based nutritional interventions and bone-related outcomes in adolescent athletes.

Design	Sample size	Randomization	Blinding	Comparator	Outcome assessment	Adherence	Main risk	Overall quality	Follow-up	Study
Pre–post pilot	5	No/Not applicable	Not applicable/NR	None	Generally structured/objective	NR	Very small uncontrolled clinical sample	Low/Poor	12 wk	(59)
Parallel-group controlled trial	54 analyzed (60 recruited)	Stratified allocation by age, sex, and body mass; randomization method not explicitly described	Double-blind; participants and investigators blinded to beverage identity	Isoenergetic carbohydrate (CHO) and non-nutritive water (H_2_O) controls	Objective performance testing and laboratory biomarkers; muscle soreness assessed by self-report	Not clearly reported; beverage consumption was administered under study supervision	Small sample; exclusion of 6 participants from the original cohort; short 24-h follow-up; dinner not standardized; randomization procedure and adherence reporting insufficiently detailed	Moderate	24 h after baseline/HIIS	(57)
Double-blind, placebo-controlled, parallel-group intervention	*n* = 58 (PRO = 21, CHO = 19, H_2_O = 18)	Stratified allocation matched for age, body mass, and sex; randomization method not explicitly described	Double-blind; participants and investigators directly involved were blinded to beverage contents; independent assistant prepared/pre-packaged beverages	Isoenergetic carbohydrate (maltodextrin) and non-nutritive flavored water	Objective biochemical assessment of CTX-I and PINP measured in duplicate; estradiol also assessed; repeated-measures assessment at baseline, 8 h, and 24 h	Not explicitly quantified; supplement administration was provided by the study team	Very short follow-up; small sample; unclear randomization procedure; missing values and extensive outlier replacement/trimming; bone turnover indices were indirect biomarkers rather than direct tissue-level outcomes	Moderate	8 h and 24 h	(55)
Randomized controlled, parallel-group trial; 16-week supplementation following an 8-week control period	50 recruited; 47 completed and analyzed (GY: *n* = 24; WP: *n* = 23)	Computer-generated 1:1 randomization; stratified by sex	Single-blind; investigators blinded, participants aware of supplement type	Isonitrogenous/ isoenergetic whey protein (WP) vs. Greek yogurt (GY)	Objective laboratory assessment of bone turnover and inflammatory biomarkers (P1NP, CTX-I, OC, OPG, SOST, RANKL, IGF-1, IL-1β, TNF-α, IL-6, IL-10); blood collected at weeks 0, 8, 16, and 24	Moderate; self-reported 10-point adherence scores, approximately 7.1–8.7/10; 3 participants withdrew because of difficulty maintaining supplementation	Participant unblinding and self-reported adherence; 23/188 blood samples missing; several biomarker outliers excluded; small sample size	Moderate–high	24 weeks (8-week control + 16-week intervention)	(56)

CHO, carbohydrate; CTX-I, C-terminal telopeptide of type I collagen; GY, Greek yogurt; HIIS, high-intensity interval swimming; H_2_O, water; IL, interleukin; OC, osteocalcin; OPG, osteoprotegerin; P1NP, procollagen type I N-terminal propeptide; PRO, protein; RANKL, receptor activator of nuclear factor κB ligand; SOST, sclerostin; TNF-α, tumor necrosis factor-alpha; WP, whey protein.

**TABLE 3:** Effects of creatine supplementation combined with exercise on performance, rehabilitation, and body composition in adolescent athletes.

Population	Intervention	Study design	Key outcomes measured	Main results	Conclusion	Study
*n* = 18 adolescent fin swimmers (injured)	Creatine vs. placebo during rehabilitation	Randomized, double-blind, placebo-controlled	Segmental lean mass (SLM), plantar flexion torque (PFT), pain (NRS), CK levels	↓ muscle loss during immobilization; ↑ recovery of strength (PFT); ↓ pain; ↓ CK levels in CR vs. PL	Creatine supports faster and more effective tendon injury rehabilitation	(79)
*n* = 19 elite male soccer players (∼17 yrs)	Creatine (0.03 g/kg/day, 14 days) vs. placebo	Randomized, double-blind, placebo-controlled	Peak power (PPO), mean power (MPO), fatigue index (FI), total work (Wingate test)	↑ PPO, MPO, and total work with creatine; no change in FI	Short-term low-dose creatine improves muscle power output	(69)
*n* = 28 young soccer players	Creatine ± caffeine + variable resistance (I-sVR)	Randomized, double-blind, placebo-controlled	Repeated sprint ability (RSA), half-back squat (HBS) performance	No significant differences between groups	Combined ergogenic aids show no additive performance benefit	(75)
*n* = 16 male junior fin swimmers (∼15.9 yrs)	Creatine (20 g/day for 5 days) vs. placebo	Randomized, double-blind	Power output (Bosco test), swimming performance (100 m), lactate	↑ Power output ( + 20%); ↓ swimming time; improved repeated swim performance; ↓ lactate	Short-term creatine enhances anaerobic performance and dynamic strength	(70)
*n* = 16 junior female swimmers	Creatine (20 g/day for 21 days) vs. placebo	Randomized, double-blind	Swimming velocity, hydrodynamics (drag force, Cx), power, body composition	↓ drag force and hydrodynamic variables (∼25%); no change in performance or body composition	Creatine improves swimming efficiency but not performance or body composition	(76)
*n* = 20 junior swimmers (male and female; ∼16.4 yrs)	Creatine (loading + maintenance for 4 weeks) vs. placebo	Controlled trial	Sprint performance (50 m, 100 m), swim bench work, body composition, lactate	No improvement in swim sprint performance; ↑ swim bench total work; no body composition changes	Creatine enhances dry-land performance but not in-water sprint performance	(77)
*n* = 18 junior swimmers (male and female)	Creatine (21 g/day, 9 days) vs. placebo	Randomized, double-blind	Repeated 100 m swim performance, arm ergometer sprints	↓ swim time (especially early sprints); improved cumulative performance	Creatine provides ergogenic benefits for repeated sprint performance	(71)
*n* = 18 junior women wrestlers (∼18.7 yrs)	Creatine (10 g/day) + strength training vs. training alone vs. control (6 weeks)	Randomized controlled trial	Strength (1RM), agility, power, BMI, hypertrophy	Significant improvements in strength, power, agility, and hypertrophy only in creatine group	Creatine + training enhances physical fitness and muscle hypertrophy	(74)
*n* = 23 U16 male basketball players (∼14.3 yrs)	Creatine (0.1 g/kg/day) + strength and plyometric training (8 weeks)	Randomized controlled trial	Jump performance (SJ, DJ, CMJ, ABK), game performance (points, minutes)	↑ ABK jump power; ↑ scoring performance; limited effects on other jumps	Creatine improves specific power output and sport performance	(72)
*n* = 14 male elite soccer players	Creatine vs. placebo during 7-week pre-season training	Randomized, double-blind, placebo-controlled	Countermovement jump (lower-limb power)	No significant improvement; creatine prevented decline in performance vs. placebo	Creatine helps maintain muscle power during intense training periods	(78)
*n* = 20 young male soccer players (∼16.6 yrs)	Creatine (30 g/day for 7 days) vs. placebo	Randomized controlled trial	Dribbling, sprint power, endurance, vertical jump	↑ Dribbling, sprint power, vertical jump; no change in endurance	Creatine improves soccer-specific skills and explosive performance	(73)

CR, creatine group; PL, placebo; SLM, segmental lean mass; PFT, plantar flexion torque; NRS, numeric rating scale; CK, creatine kinase; PPO, peak power output; MPO, mean power output; FI, fatigue index; RSA, repeated sprint ability; HBS, half-back squat; I-sVR, intra-serial variable resistance; Cx, hydrodynamic coefficient; BMI, body mass index; 1RM, one-repetition maximum; SJ, squat jump; DJ, drop jump; CMJ, countermovement jump; ABK, Abalakov jump.

**TABLE 4:** Methodological characteristics and quality assessment of studies investigating the effects of creatine supplementation on physical performance and related outcomes.

Design	Sample size	Randomization	Blinding	Comparator	Outcome assessment	Adherence	Main risk	Overall quality	Follow-up	Study
Randomized, double-blind, placebo-controlled parallel-group trial	18 (CR: *n* = 9; PL: *n* = 9)	Random assignment; method of sequence generation and allocation concealment not reported	Double-blind; participants and investigators blinded to treatment	Placebo matched for taste, texture, and appearance	Objective measures of SLM, plantar-flexion torque, and CK; pain assessed using NRS; repeated measurements across rehabilitation phases	Treatment logs were maintained, but quantitative adherence was not clearly reported	Very small sample; no formal a priori power calculation; incomplete reporting of randomization/ allocation procedures; short 6-week follow-up; adherence not objectively quantified; rehabilitation and maturation may introduce variability	Moderate	6 weeks (2-week immobilization + 4-week rehabilitation)	(79)
Matched-pair, randomized, double-blind, placebo-controlled parallel-group trial	19 analyzed (Cr: *n* = 9; placebo: *n* = 10; 20 initially randomized)	Matched pairs based on muscle power output, followed by random allocation; sequence-generation and allocation-concealment procedures not reported	Double-blind; coded, visually and texturally matched supplements	Maltodextrin placebo	Objective laboratory-based Wingate measures of peak power output, mean power output, fatigue index, and total work; standardized testing with high reported test–retest reliability	Excellent; > 99% adherence based on return of empty supplement packages	Very small sample; one participant withdrew; baseline body weight differed between groups; short intervention; no adjustment for baseline weight imbalance; limited dietary control; no significant group × time effects for most outcomes despite within-group improvements	Moderate–high	14 days	(69)
2 × 2 factorial, double-blind, placebo-controlled intervention trial	28 (4 groups; n = 7/group)	Sample-matched allocation based on age and 1RM; specific randomization sequence/concealment not clearly reported	Double-blind; creatine and caffeine/placebo preparations were administered in matched protocols	Creatine vs. maltodextrin placebo; caffeine vs. maltodextrin placebo, with factorial assessment of combined effects	Objective performance testing using repeated-sprint ability (RSA) and half-back squat (HBS) velocity/power; standardized photocell and linear-encoder measurements	Not quantitatively reported; participants were instructed to follow supplementation protocols and maintain the club-prescribed diet	Very small groups; unclear allocation concealment; no formal adherence quantification; no specific dietary monitoring; multiple factorial comparisons; short intervention; potential confounding from the combined caffeine, creatine, and I-sVR protocols	Low–moderate	14 days of creatine supplementation; acute post-supplementation performance assessment	(75)
Quasi-experimental, 3-group parallel design with pre-, mid-, and post-testing	18 female junior freestyle wrestlers (21 recruited; 3 withdrew)	Participants were randomly assigned to 3 groups; however, the randomization method is insufficiently described	Not reported	Creatine + strength training vs. strength training alone vs. no training/no creatine control	Body composition (weight, BMI, BF%, FFM), VO2max, resting HR, 1RM chest/leg press, Illinois Agility Test, vertical jump/muscular power, and muscle circumference/hypertrophy; assessed at baseline, 3 weeks, and 6 weeks	Not clearly reported; training was supervised, but creatine supplementation compliance was not quantified	High risk: quasi-experimental/purposive recruitment, very small sample, no reported blinding, unclear allocation concealment and supplement adherence, and baseline comparability is not fully established	Low	6 weeks (pretest ↑ midtest at 3 weeks ↑ posttest at 6 weeks)	(74)
Randomized controlled, pretest–posttest study; 8-week intervention	23 completed (CrM *n* = 12; CON *n* = 11)	1:1 random allocation using	Not blinded; non-placebo control	Non-placebo control receiving the same resistance/plyometric training without creatine	Objective, validated jump-performance tests (SJ, DJ, CMJ, ABJ); basketball performance (PPG, MPG) obtained from official federation records	Not explicitly quantified; supplementation on non-training days was monitored through daily-dose provision/personal communication	Lack of placebo/blinding; small sample; one dropout; possible performance/expectancy bias	Moderate	8-week intervention; basketball performance additionally assessed during 6 weeks pre-intervention, 6 weeks during intervention, and 6 weeks post-intervention	(72)
Randomized, double-blind, placebo-controlled parallel-group trial; 7-week pre-season intervention	14 completed (CrM *n* = 7; placebo *n* = 7); 23 initially recruited	Not explicitly described	Double-blind; coded supplements, with investigators and participants unaware of allocation; blinding checked	Dextrose placebo + identical training and dietary schedules	Objective CMJ assessment using a strain-gauge force plate at 1,000 Hz; dietary intake and anthropometry assessed pre/post	Weekly personal communication; supplement purity verified by HPLC (99.9%)	Very small final sample; substantial attrition (9/23); five players discharged, three injured, one refused supplementation; no direct muscle creatine measurement; external resistance-training load not recorded	Moderate-to-high methodological quality, but limited by very small sample and attrition	7 weeks during pre-season; outcomes assessed before and after supplementation	(78)

Cr, creatine; CrM, creatine monohydrate; PL, placebo; CON, control; SLM, skeletal lean mass; CK, creatine kinase; NRS, numerical rating scale; 1RM, one-repetition maximum; RSA, repeated sprint ability; HBS, half-back squat; BF%, body fat percentage; FFM, fat-free mass; VO2max, maximal oxygen uptake; HR, heart rate; SJ, squat jump; DJ, drop jump; CMJ, countermovement jump; ABJ, Abalakov jump; PPG, points per game; MPG, minutes per game; I-sVR, intra-series variable resistance; RPE, rating of perceived exertion.

**TABLE 5:** Major immunometabolic mediators influenced by aerobic exercise, whey protein, and creatine in adolescents.

Population	Intervention/ Condition	Immunometabolic mediators assessed	Main findings	Physiological implications	Study
Elite athletes (*n* = 80; < 25 and > 25 years) participating in low-, moderate-, and high-intensity sports	Comparison according to sport intensity	IL-6, IL-8, IL-10, TNF-α, catalase activity, telomere length	↑ IL-10 (high-intensity sports); ↑ IL-6 (older high-intensity athletes); ↑ telomere length with sport intensity; ↔ positive correlations among IL-6, IL-8, IL-10, and TNF-α	Enhanced immunometabolic adaptation	(108)
Male adolescent swimmers (*n* = 9; 16–19 years)	Seasonal swim training (extensive, intensive, and tapering phases)	IL-6, IL-10, TNF-α, IGF-I, IGFBP-3	↑ IL-10 and IGF-I during tapering; ↓ IL-6 associated with ↑ IGF-I during intensive training	Improved training monitoring and recovery	(82)
Adolescent swimmers (*n* = 54; 11–17 years)	Post-exercise whey protein vs. isoenergetic carbohydrate vs. placebo following high-intensity interval swimming	IL-6, IL-10, TNF-α, creatine kinase (CK)	↑ IL-10 following whey protein; ↔ IL-6, TNF-α, CK, and performance outcomes	Enhanced anti-inflammatory recovery response	(57)
Adolescent athletes (*n* = 47; 15–17 years)	Greek yogurt vs. whey protein supplementation during a 16-week competitive season	IL-1β, IL-6, IL-10, TNF-α, osteocalcin, osteoprotegerin (OPG), PINP, CTX-I, sclerostin, IGF-I	↓ IL-6 in the Greek yogurt group ↑↑ IL-1β in the whey protein group ↑ sex-specific changes in inflammatory and bone biomarkers ↑ fluctuations in OPG, osteocalcin, CTX-I, and sclerostin	Protein intake, irrespective of source, may modulate bone–immune crosstalk during adolescence	(56)
Male soccer players under 20 years (*n* = 25)	Creatine supplementation (0.3 g/kg/day for 7 days) combined with repeated-sprint exercise	TNF-α, CRP, malondialdehyde, glutathione, LDH, catalase, superoxide dismutase	↓ TNF-α following creatine supplementation ↑↓ CRP following creatine supplementation ↑↓ LDH response ↑↔ oxidative stress marker elevations	Creatine may reduce exercise-induced inflammation and improve recovery during high-intensity exercise	(83)

IL, interleukin; TNF-α, tumor necrosis factor-alpha; IGF-I, insulin-like growth factor-I; IGFBP-3, insulin-like growth factor-binding protein-3; CK, creatine kinase; CRP, C-reactive protein; LDH, lactate dehydrogenase; PINP, procollagen type I N-terminal propeptide; CTX-I, C-terminal telopeptide of type I collagen; OPG, osteoprotegerin.

**TABLE 6:** Major signaling molecules involved in muscle–bone–brain crosstalk in response to exercise and nutritional interventions: evidence from adolescent, adult, and preclinical studies.

Signaling molecule/pathway	Experimental model	Main findings	Interpretation and physiological relevance	Study
PGC-1α/FNDC5/Irisin/BDNF axis	Mouse models, primary cortical neurons, and in vivo peripheral FNDC5 overexpression	↑ Exercise-induced FNDC5 expression in the hippocampus; ↑ PGC-1α-mediated neuronal FNDC5 expression; ↑ BDNF and neuroprotective gene expression following FNDC5 overexpression	Provides preclinical evidence that the PGC-1α/FNDC5/irisin/BDNF pathway may contribute to exercise-related neuroplasticity; direct evidence in adolescents remains limited	(18)
PGC-1α-induced FNDC5/Irisin signaling	Mouse skeletal muscle, adipose tissue cultures, and human exercise studies	↑ Exercise-induced PGC-1α and FNDC5 expression in skeletal muscle; ↑ circulating irisin; ↑ UCP1 expression and white-adipose-tissue browning in experimental models; ↑ energy expenditure and glucose homeostasis	Supports a potential role of PGC-1α/FNDC5/irisin signaling in metabolic adaptation and muscle–adipose communication; findings from experimental models should not be directly extrapolated to adolescents	(86)
Lactate/SIRT1/PGC-1α/FNDC5/BDNF pathway	Male mouse model with exercise and hippocampal analyses	↑ Lactate transport across the blood–brain barrier; ↑ SIRT1 activation; ↑ PGC-1α/FNDC5/BDNF signaling; ↑ learning and memory performance	Provides mechanistic evidence linking exercise-related lactate signaling with brain plasticity in mice; whether this pathway operates similarly during human adolescence remains to be established	(89)
Undercarboxylated osteocalcin (ucOC) signaling	Post-hoc analysis of older women ( > 70 years) from a randomized placebo-controlled trial	↑ Relative ucOC associated with muscle strength; ↓ ucOC associated with ↑α-ACT concentrations	Suggests a potential muscle–bone endocrine relationship in older adults; the relevance of these findings to adolescent musculoskeletal development remains uncertain	(92)
Irisin-mediated osteoblast differentiation	Mouse myoblast cultures, exercised mice, and bone marrow stromal cells	↑ Exercise-related irisin; ↑ osteoblast differentiation; ↑ alkaline phosphatase and collagen I expression	Provides preclinical evidence for muscle-to-bone endocrine signaling through irisin; direct confirmation of this mechanism in adolescents is currently lacking	(90)
Protein intake–bone–immune interaction (Greek yogurt vs. whey protein)	Adolescent athletes during a 16-week competitive season; Greek yogurt versus whey protein	↔/modest changes in osteocalcin, OPG, CTX-I, sclerostin, IL-1β, IL-6, IL-10, and TNF-α, with sex-specific differences	Provides direct adolescent evidence that protein intake may influence bone-remodeling and inflammatory biomarkers, although the effects were modest and variable	(56)
Whey protein and bone turnover responses to exercise	Adolescent swimmers following high-intensity interval swimming	↑ IL-10; ↔ IL-6, TNF-α, and performance outcomes	Provides direct adolescent evidence that whey protein may influence selected inflammatory responses during recovery; broader effects on muscle–bone–brain crosstalk remain uncertain	(57)

α-ACT, alpha-1-antichymotrypsin; BDNF, brain-derived neurotrophic factor; CTX-I, C-terminal telopeptide of type I collagen; FNDC5, fibronectin type III domain-containing protein 5; IL, interleukin; OPG, osteoprotegerin; PGC-1α, peroxisome proliferator-activated receptor gamma coactivator-1 alpha; SIRT1, sirtuin 1; TNF-α, tumor necrosis factor-alpha; UCP1, uncoupling protein 1; ucOC, undercarboxylated osteocalcin.

**TABLE 7:** Effects of aerobic exercise, whey protein, and creatine on motor competence-related outcomes in adolescents.

Population	Intervention	Motor competence-related outcomes measured	Main results	Implications for motor competence	Study
*n* = 20 young male soccer players (16.6 ± 1.9 yrs)	Creatine monohydrate (30 g/day; 7 days) vs. placebo	Dribbling skill, sprint-power test, vertical jump, endurance performance	↓ dribbling time; ↑ sprint power; ↑ vertical jump height; no effect on endurance	Creatine improved selected soccer-specific performance and explosive motor performance.	(73)
*n* = 16 elite male junior fin swimmers (15.9 ± 1.6 yrs)	Creatine monohydrate (20 g/day; 5 days) vs. placebo	Mechanical power output (Bosco test), repeated swim performance	↑ dynamic strength ( + 20.2%); ↓ swimming time during repeated maximal swims	Creatine improved anaerobic motor performance and repeated sport-specific skills	(70)
*n* = 14 elite male soccer players	Creatine monohydrate during 7-week pre-season training vs. placebo	Countermovement jump (CMJ) performance	Creatine prevented reductions in jump performance during intensive training	Creatine may preserve neuromuscular performance during periods of high training load	(78)
*n* = 23 U16 male basketball players (14.3 ± 0.4 yrs)	Creatine monohydrate (0.1 g/kg/day) + strength and plyometric training (8 weeks)	Squat jump (SJ), drop jump (DJ), countermovement jump (CMJ), Abalakov jump (ABK), game performance	Significant improvement in ABK performance and points scored per game; no additional effects on SJ, DJ, or CMJ	Creatine combined with complex training enhanced explosive power and sport-specific performance	(72)
*n* = 22 adolescent soccer players (15.6 ± 0.2 yrs; 59% female)	Whey protein (20 g twice daily) vs. isocaloric placebo for 10 weeks	V̇O_2_max, sprint time, muscle strength, muscular endurance	↑ V̇O_2_max; no additional improvements in sprint performance or strength	Whey protein may indirectly support motor competence by enhancing aerobic fitness	(58)
*n* = 54 swimmers (11–17 yrs)	Post-exercise whey protein vs. carbohydrate vs. water	Recovery indices, muscle soreness, subsequent performance	↓ muscle soreness; no improvement in swimming performance	Whey protein may facilitate recovery and readiness for subsequent motor practice sessions	(57)
*n* = 80 adolescent and adult elite athletes	Participation in sports of different intensities (low-, moderate-, and high-intensity sports)	Cytokine profile, oxidative stress, telomere length	High-intensity athletes exhibited longer telomeres and distinct immunological adaptations	Regular intensive exercise may support long-term physiological adaptations underpinning motor development	(103)
*n* = 80 adolescents (15–18 yrs)	Acute endurance, strength, or coordination exercise (25 min)	Attention performance (d2 test)	All exercise modalities improved attention compared with control	Acute exercise enhances cognitive processes associated with motor learning and skill acquisition	(105)
*n* = 40 children participating in physical education	Integrative neuromuscular training (INT) twice weekly for 8 weeks	Long jump, single-leg hop, running performance, muscular fitness	Significant improvements in health- and skill-related fitness measures	Integrative neuromuscular training effectively enhances foundational motor competence	(109)

ABK, Abalakov jump; CMJ, countermovement jump; DJ, drop jump; INT, integrative neuromuscular training; SJ, squat jump; U16, under 16 years of age; V̇O_2_max, maximal oxygen uptake.

**TABLE 8:** Practical considerations for whey protein and creatine supplementation in adolescents.

Supplement	Practical dosing consideration	Evidence-informed range/protocol	Adolescent-specific considerations
Whey protein	Daily protein intake	Approximately 1.4–2.0 g/kg/day total protein, depending on training load, growth demands, and habitual dietary intake	Supplements should complement rather than replace a balanced diet; individual requirements should account for growth, energy availability, training volume, and dietary protein intake
Whey protein	Post-exercise intake	Approximately 0.25–0.4 g/kg per serving when dietary protein intake is inadequate or when a practical post-exercise protein source is needed	No evidence supports mandatory post-exercise supplementation when adequate dietary protein is already consumed
Creatine	Loading phase	Approximately 0.3 g/kg/day for 5–7 days, divided into several doses	A loading phase is not essential; because adolescent-specific evidence is limited, unnecessary high-dose or prolonged loading should be avoided
Creatine	Maintenance	Approximately 0.03–0.1 g/kg/day	Use should be individualized and preferably supervised; long-term safety data in adolescents remain limited
Creatine	General safety consideration	No universally established adolescent-specific upper safe dose	Particular caution is warranted in adolescents with renal/hepatic disease, dehydration, concomitant nephrotoxic medications, or other relevant medical conditions

## 3 Energy metabolism and exercise performance in adolescents

Energy metabolism in adolescents is shaped by the interaction between ongoing growth, biological maturation, and exercise-induced metabolic adaptations. During this developmental stage, metabolic pathways exhibit substantial plasticity, facilitating responses to diverse physical demands. Both aerobic and anaerobic energy systems contribute to ATP production, with their relative contributions varying according to exercise intensity, duration, and training status. Adolescents generally demonstrate a greater reliance on oxidative metabolism and a lower glycolytic response than adults, which is associated, in part, with differences in glycolytic enzyme activity and lactate production (24, 25). Nevertheless, as biological maturation progresses and systematic training is undertaken, anaerobic capacity increases, supporting performance during high-intensity activities. Hormonal changes during puberty, including alterations in growth hormone, testosterone, and insulin-like growth factor 1 (IGF-1), also influence muscle development, substrate utilization, and exercise capacity (26). These developmental characteristics highlight the importance of considering biological maturation when interpreting metabolic responses to exercise and designing training and nutritional strategies for adolescent athletes (27).

### 3.1 ATP–phosphocreatine (ATP–PCr) system and high-intensity performance demands

The ATP–PCr system is fundamentally important for short-duration, high-intensity activities commonly encountered in youth sports, including sprinting, jumping, and rapid changes of direction. It provides a rapid source of ATP through the phosphocreatine-mediated resynthesis of ATP within skeletal muscle, allowing high rates of energy provision during the initial seconds of intense exercise (28). The functional capacity of this system is influenced by muscle mass, intramuscular creatine and phosphocreatine availability, and the activity of enzymes involved in phosphagen metabolism, which change with growth and maturation (29). Although younger athletes may have lower absolute phosphocreatine stores than adults, they can demonstrate relatively rapid phosphocreatine resynthesis during recovery between repeated high-intensity efforts (30). This developmental difference may contribute to the characteristic ability of younger athletes to recover relatively quickly from brief maximal efforts, although performance responses remain dependent on maturation, training status, and exercise modality. Sprint and resistance training can increase the functional capacity of the phosphagen system and improve performance during repeated high-intensity exercise. The potential contribution of creatine supplementation to phosphocreatine availability and high-intensity performance is considered separately in the later sections of this review.

### 3.2 Aerobic vs. anaerobic energy contributions in youth sports

Youth sports generally involve intermittent activity patterns that require coordinated contributions from both aerobic and anaerobic energy systems. Aerobic metabolism predominates during prolonged or moderate-intensity exercise and also contributes substantially to recovery between repeated high-intensity efforts by supporting oxidative ATP production and substrate utilization (31). In contrast, anaerobic glycolysis and the ATP–PCr system provide a greater proportion of energy during brief, high-intensity efforts (32). Compared with adults, adolescents typically exhibit greater relative reliance on oxidative metabolism and a lower glycolytic contribution during intense exercise, accompanied by lower blood lactate accumulation under comparable conditions (32). These characteristics may favor efficient aerobic energy provision and recovery but can constrain the absolute capacity for anaerobic power in less mature individuals. With advancing biological maturation and systematic training, the relative contribution and capacity of anaerobic metabolism increase, contributing to improved performance in sprinting, jumping, and other high-intensity activities (33, 34). Accordingly, adolescent performance should be interpreted as the product of interacting aerobic and anaerobic systems rather than as a consequence of a single dominant metabolic pathway.

### 3.3 Developmental considerations in adolescent metabolism

Adolescent metabolism is strongly influenced by ongoing growth, biological maturation, and endocrine changes that affect energy production, substrate utilization, and exercise capacity (35). During puberty, increases in sex hormones and growth-related factors accompany changes in muscle mass, body composition, cardiovascular function, and metabolic enzyme activity, contributing to progressive improvements in both aerobic and anaerobic exercise capacity (36). Importantly, these adaptations are not synchronized across individuals and cannot be inferred reliably from chronological age alone. Consequently, adolescents of the same chronological age may exhibit markedly different metabolic responses because of differences in biological maturation, training status, and body composition. Substrate utilization also changes according to exercise intensity, nutritional status, and training background, with carbohydrate oxidation becoming increasingly important as exercise intensity rises and lipid oxidation contributing more substantially during lower-intensity or prolonged activity (37). Adequate energy availability is therefore essential for supporting both exercise demands and the energetic requirements of growth. In adolescent athletes, training and nutritional strategies should consequently be individualized according to biological maturation, training load, habitual dietary intake, and growth-related requirements (4).

### 3.4 Mitochondrial bioenergetics and metabolic flexibility during adolescence

Adolescence is accompanied by substantial physiological and metabolic changes that support somatic growth, maturation, and increasing physical demands. Mitochondrial bioenergetics is particularly relevant because mitochondria provide the major cellular site for oxidative phosphorylation and ATP production, thereby supporting skeletal muscle function and whole-body energy metabolism (14). Changes in muscle mass, training status, and endocrine environment during adolescence may influence mitochondrial oxidative capacity and substrate utilization; however, the magnitude and timing of these adaptations vary among individuals according to maturation and exercise history (38). Metabolic flexibility refers to the capacity to adjust the relative oxidation of carbohydrate and lipid substrates according to energetic requirements, exercise intensity, and nutritional conditions (39). Regular aerobic exercise can enhance oxidative capacity through adaptations involving mitochondrial enzymes, substrate utilization, and fatty-acid oxidation (15). These adaptations may contribute to the ability of adolescent athletes to sustain prolonged exercise and recover between training bouts. Exercise-induced mitochondrial adaptations are mediated by coordinated transcriptional and metabolic responses, including pathways involved in mitochondrial biogenesis and oxidative metabolism (40). Nevertheless, much of the mechanistic evidence describing these processes derives from adult or experimental models, and the extent to which specific molecular adaptations differ during adolescence remains incompletely characterized. Therefore, mitochondrial adaptations in adolescent athletes should be interpreted in the context of biological maturation rather than assumed to follow the same trajectory as those observed in adults. Overall, mitochondrial oxidative capacity and metabolic flexibility represent important components of adolescent exercise adaptation, but their responses are influenced by maturation, training characteristics, and energy availability.

### 3.5 Exercise-induced activation of AMPK–PGC-1α signaling pathways

Exercise-induced skeletal muscle adaptation is regulated by an interconnected network of intracellular signaling pathways, among which the AMPK–PGC-1α axis plays an important role in linking cellular energy status with mitochondrial adaptation. During aerobic exercise, increased ATP turnover and alterations in cellular adenine nucleotide balance can activate AMPK, an energy-sensing serine/threonine kinase that promotes pathways involved in ATP production while suppressing selected energy-consuming processes (41). AMPK can subsequently influence PGC-1α activity, thereby contributing to transcriptional programs associated with mitochondrial biogenesis and oxidative metabolism (42). PGC-1α is a transcriptional coactivator that coordinates the expression of genes involved in mitochondrial function, oxidative phosphorylation, fatty-acid oxidation, and other aspects of skeletal muscle metabolic adaptation (43). Rather than functioning as an isolated regulator, PGC-1α operates within a broader signaling network that integrates cellular energy status, exercise-induced stress, and transcriptional responses. Repeated aerobic exercise can therefore promote adaptations in oxidative capacity through coordinated AMPK–PGC-1α signaling and related pathways (42, 44). During adolescence, these pathways operate within a developing metabolic system, and their responses may vary according to biological maturation, exercise intensity, training status, and energy availability. Although regular aerobic exercise is associated with enhanced oxidative adaptations, direct adolescent-specific evidence for every component of the AMPK–PGC-1α pathway remains limited. The potential downstream links between PGC-1α, fibronectin type III domain-containing protein 5 (FNDC5)/irisin, and exercise-induced neuroplasticity are discussed separately in the muscle–bone–brain crosstalk section to avoid duplication. Similarly, the interaction between exercise-induced energy sensing and nutritional signaling is considered in the subsequent sections addressing whey protein and creatine (Figure 1).

**FIGURE 1:**
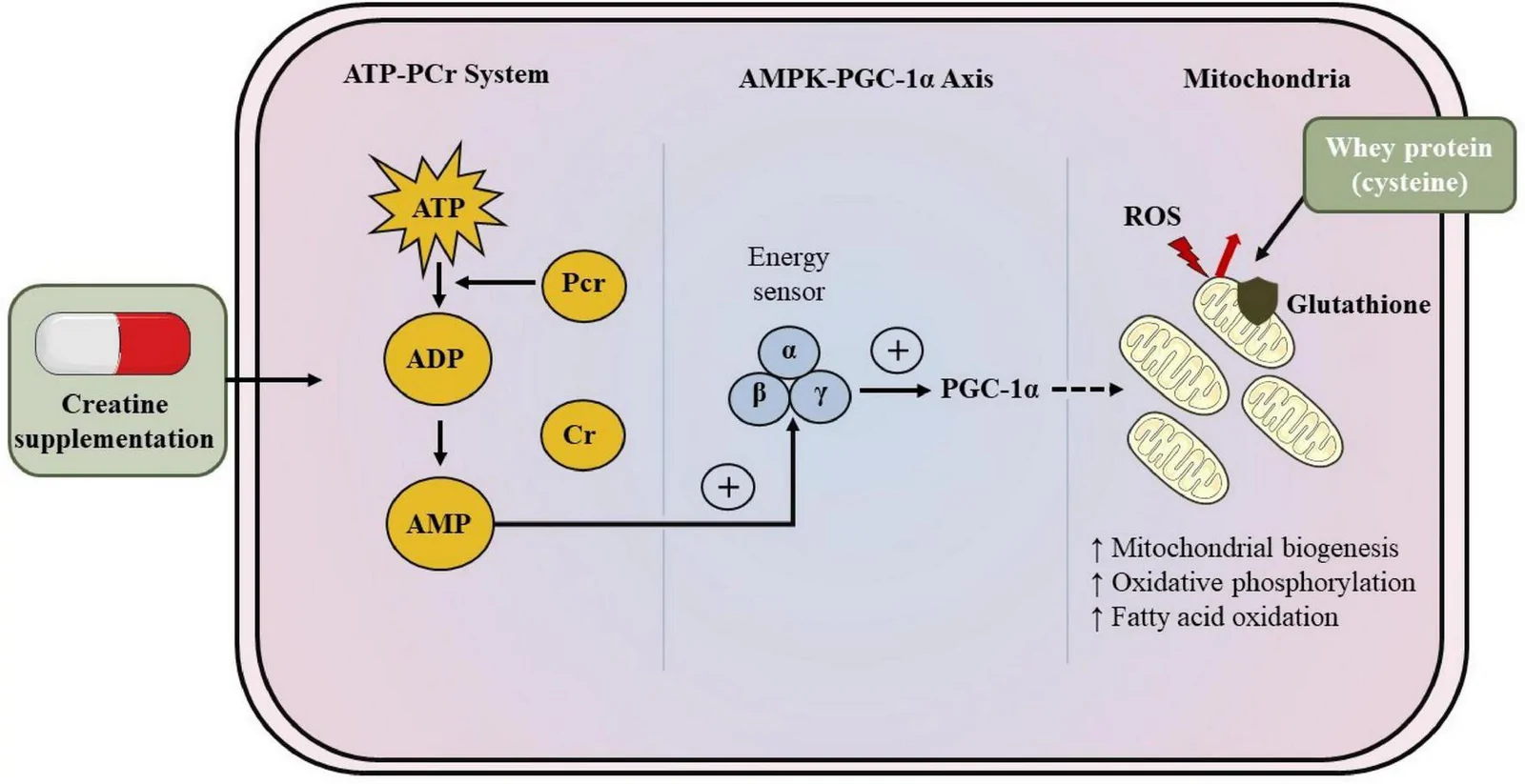
Energy dynamics and the AMPK-PGC-1α signaling network in skeletal muscle. This schematic illustrates the cellular bioenergetics of exercise and the synergistic role of nutritional interventions. During high-intensity exercise, creatine supplementation augments phosphocreatine (PCr) reserves to accelerate ATP resynthesis. During aerobic exercise, an increased AMP/ATP ratio activates the AMPK–PGC-1α axis, driving mitochondrial biogenesis and enhancing oxidative capacity. Additionally, whey protein supplies cysteine for glutathione (GSH) synthesis, which helps buffer the reactive oxygen species (ROS) generated by increased mitochondrial activity.

## 4 Whey protein supplementation and exercise adaptations

Whey protein supplementation is widely acknowledged as a nutritional strategy that may support exercise-related adaptations, particularly during the adolescent phase—a developmental stage characterized by rapid growth and increased anabolic demands. Abundant in essential amino acids and leucine, whey protein can stimulate MPS and support tissue remodeling. When integrated with structured exercise, whey protein may support recovery and the maintenance or development of lean tissue, although effects on performance outcomes in adolescents remain inconsistent. At the molecular level, whey protein provides amino acids, particularly leucine, that can stimulate mTOR complex 1 (mTORC1)-dependent anabolic signaling and increase amino acid availability (45–47). These mechanisms provide biological plausibility for interactions between protein intake and exercise adaptation; however, direct evidence for such molecular effects and their long-term functional consequences in adolescents remains limited. Accordingly, the combination of whey protein and exercise should be viewed as a potentially supportive nutritional strategy rather than a universally ergogenic intervention.

### 4.1 Muscle strength, hypertrophy, and body composition

Whey protein supplementation may support muscle-related adaptations when combined with exercise training, particularly through its provision of essential amino acids and leucine. Its rapid digestibility and elevated leucine content facilitate stimulation of MPS through mTORC1-related signaling, thereby providing a mechanistic basis for supporting myofibrillar protein accretion (6, 48, 49). However, these molecular responses should not be equated with consistent improvements in strength, hypertrophy, or body composition in adolescent athletes. Available clinical evidence is limited and heterogeneous, and some studies report improvements in selected outcomes without demonstrating additional benefits across all measures of physical performance or body composition. Therefore, the anabolic mechanisms of whey protein provide a plausible basis for supporting adaptation, but the magnitude and consistency of functional benefits during adolescence remain uncertain.

### 4.2 Recovery, muscle damage, and inflammatory responses

Whey protein supplementation may influence selected aspects of post-exercise recovery and inflammatory regulation. Following exercise-induced muscle stress, whey protein provides rapidly available essential amino acids that can support tissue repair and muscle protein turnover. In adolescent swimmers, post-exercise whey protein has been associated with reduced muscle soreness and increased IL-10, whereas other outcomes, including IL-6, tumor necrosis factor-alpha (TNF-α), creatine kinase, and subsequent performance, have shown limited or no additional changes compared with control conditions (50–52). These findings suggest that whey protein may influence selected inflammatory or perceptual markers of recovery without necessarily producing broad improvements in biochemical recovery or subsequent performance. In adolescents, effective recovery is particularly important for maintaining training quality and accommodating repeated exercise exposure; however, the available evidence does not yet establish whey protein as a consistently superior recovery intervention relative to adequate carbohydrate or total dietary protein intake.

### 4.3 Bone turnover and growth-related adaptations

Whey protein supplementation has the potential to influence bone-remodeling responses, particularly during adolescence, when substantial skeletal development occurs. Whey protein provides high-quality protein and may contribute to adequate intake of nutrients relevant to skeletal health (53, 54). Physical exercise, particularly weight-bearing activities, provides mechanical stimuli that promote bone adaptation. In adolescent swimmers, available clinical evidence indicates modest and variable effects on bone-turnover markers following post-exercise protein supplementation (55). For example, post-exercise protein supplementation was associated with a reduction in C-terminal telopeptide of type I collagen (CTX-I) at 24 h without a significant change in N-terminal propeptide of type I procollagen (PINP), accompanied by an increase in bone turnover rate (55). In addition, a 16-week comparison of Greek yogurt and whey protein in adolescent athletes identified modest, sex-specific, and transient changes in selected bone markers, including CTX-I (56). These findings provide preliminary evidence that protein intake may modulate aspects of bone remodeling during adolescence, but they do not establish improvements in bone mineral density, structural integrity, or long-term skeletal outcomes. Therefore, the potential contribution of adequate protein intake to skeletal development should be distinguished from claims of a specific bone-protective effect of whey supplementation.

### 4.4 Evidence from clinical trials in adolescents

Evidence from clinical studies suggests that the effects of whey protein supplementation in adolescents are highly context-dependent and influenced by training modality, maturational status, baseline nutritional intake, supplementation protocol, and the outcome assessed. Across the available studies summarized in Table 1, the most reproducible findings concern selected recovery and biomarker responses rather than broad improvements in athletic performance. Post-exercise whey protein was associated with reduced muscle soreness and increased IL-10 in adolescent swimmers, whereas no significant improvement was observed in subsequent swimming performance (57). Similarly, post-exercise protein supplementation was associated with changes in selected bone-turnover markers in adolescent swimmers, although these responses were modest and limited to specific outcomes (55). The 16-week Greek yogurt versus whey protein study also reported modest and sex-specific changes in selected bone and inflammatory markers, further indicating substantial variability in responses to protein supplementation (56). A recurring mechanistic rationale is that whey protein provides rapidly digestible essential amino acids, particularly leucine, which can stimulate MPS and support the anabolic response to exercise. Nevertheless, the translation of this molecular response into measurable performance benefits remains inconsistent. In adolescent soccer players, whey protein supplementation was associated with greater improvement in V̇O_2_max, whereas no additional benefits were observed for sprint performance or muscle strength (58). In adolescent swimmers, post-exercise whey protein did not improve subsequent swimming performance despite reductions in muscle soreness and an increase in IL-10 (57). These discrepancies likely reflect heterogeneity in sport-specific metabolic demands, training load, supplementation dose and timing, habitual dietary protein intake, sex distribution, and biological maturation. The evidence also suggests that the context in which supplementation is used may influence the observed response. In a pilot study involving late adolescents and young adults with Barth syndrome, resistance exercise training combined with whey protein was associated with improvements in muscle strength and quality of life, with a trend toward increased lean mass; however, the very small sample size and inclusion of participants with a specific clinical condition limit direct extrapolation to healthy adolescent athletes (59). Likewise, the bone-turnover findings in adolescent swimmers and the Greek yogurt versus whey protein study should be interpreted as preliminary evidence of changes in selected remodeling markers rather than proof of enhanced skeletal development (55, 56). From a translational perspective, the current evidence suggests that whey protein supplementation should not be considered a universal ergogenic aid for adolescent athletes. Rather, its most defensible role is as a practical source of high-quality protein that may help meet protein requirements and support selected recovery responses when dietary protein intake is inadequate or when training demands are high. Its effects should therefore be interpreted in the context of total dietary protein intake, energy availability, training status, and maturation. Nevertheless, considerable uncertainty remains because existing studies differ markedly in design and frequently involve small samples and short intervention periods, limiting definitive conclusions regarding long-term efficacy, safety, and developmental implications (Figure 2).

**FIGURE 2:**
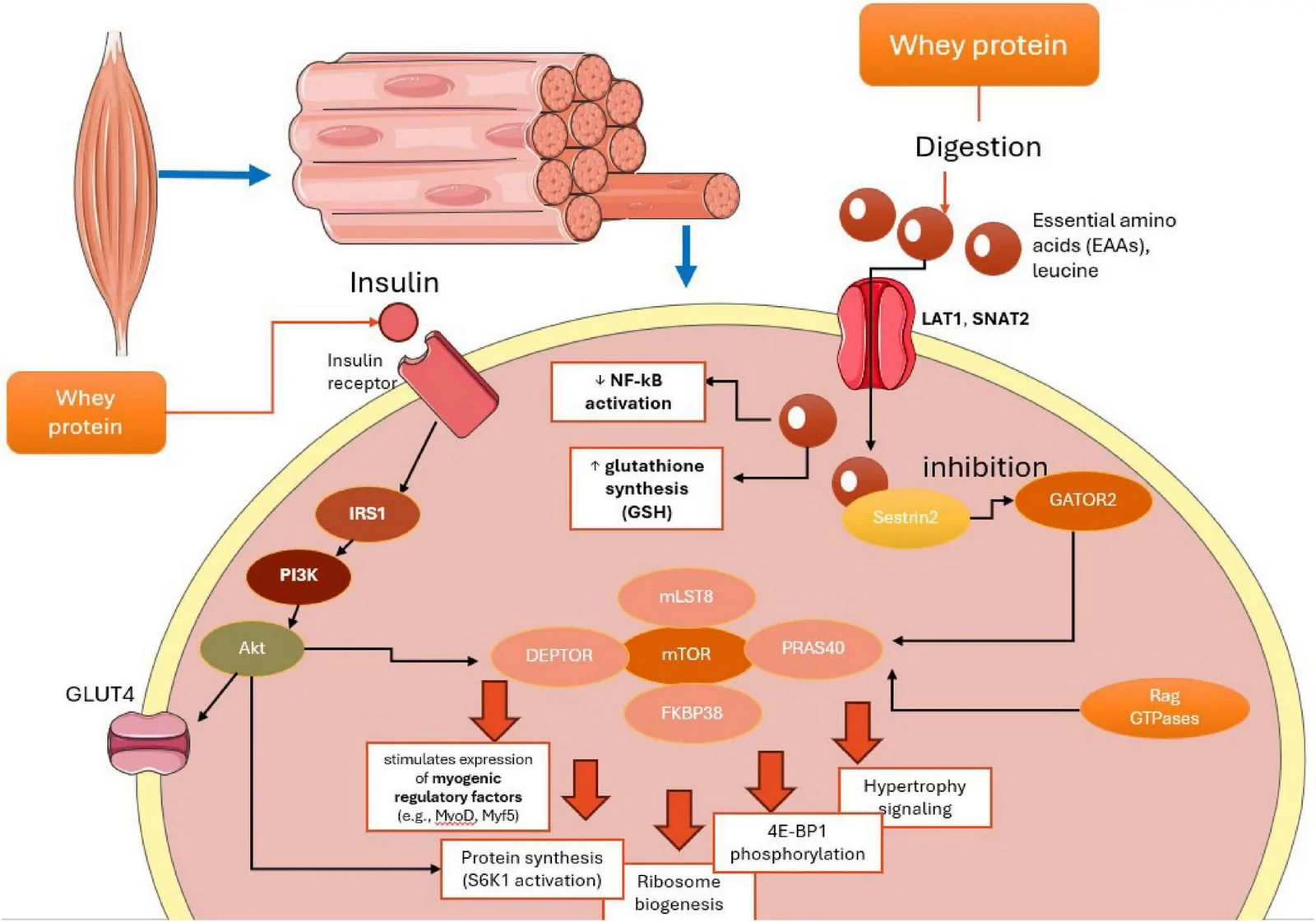
Proposed intracellular mechanisms linking whey protein supplementation to anabolic and recovery-related signaling in skeletal muscle. Following digestion, whey-derived essential amino acids, particularly leucine, increase intracellular amino acid availability in skeletal muscle through amino acid transport systems, including LAT1 and SNAT2, and may activate the leucine-sensing Sestrin2–GATOR2 pathway. This signaling can promote Rag GTPase-dependent recruitment of mTORC1 to the lysosomal surface. Concurrently, whey protein–associated increases in insulin signaling may activate the insulin receptor–IRS1–PI3K–Akt pathway, which can further support mTORC1 activation and facilitate GLUT4-mediated glucose uptake. Activated mTORC1 phosphorylates downstream targets such as S6K1 and 4E-BP1, thereby promoting translational processes involved in muscle protein synthesis and supporting anabolic remodeling. Whey-derived amino acids and bioactive components may also influence myogenic regulatory pathways and cellular redox and inflammatory responses, including glutathione synthesis and NF-κB-related signaling. Collectively, these pathways provide a plausible mechanistic framework through which whey protein may support muscle protein synthesis, tissue remodeling, and recovery following exercise. Importantly, this figure represents a proposed mechanistic model synthesized largely from established molecular and experimental evidence; the extent to which these pathways are activated by whey protein supplementation in healthy adolescent athletes remains incompletely established.

### 4.5 Critical appraisal of whey protein supplementation studies in adolescent athletes: evidence, limitations, and methodological concerns

Across the investigations summarized in Table 1, whey or dietary protein supplementation was associated with modest and outcome-specific changes in muscular, inflammatory, and osseous outcomes; however, methodological limitations reduce confidence in broad conclusions. Sample sizes were predominantly small to moderate (*n* = 5–58), with one study involving late adolescents/young adults with Barth syndrome and the remaining studies focusing primarily on adolescent athletes. Study designs and follow-up periods also varied substantially, ranging from acute post-exercise assessments to a 24-week protocol incorporating an 8-week control period and a 16-week supplementation phase. This heterogeneity limits conclusions regarding long-term adaptations, developmental safety, and sustained performance outcomes.

Several methodological concerns further affect the strength and comparability of the available evidence. Although some beverage-based trials used double-blind procedures, other studies employed single-blind designs or provided limited information regarding allocation and adherence procedures, leaving potential for performance or reporting bias. Randomization procedures were not always described in sufficient detail, particularly in some short-term trials. Differences in supplementation dose, timing, comparator conditions, exercise regimens, and habitual dietary intake further complicate cross-study comparisons. Moreover, several studies relied on relatively small samples and multiple biochemical or performance outcomes, increasing the potential for chance findings and limiting statistical precision. In some investigations, missing observations and exclusion or replacement of outlier values may have further influenced estimates. Adherence was also inconsistently quantified, ranging from supervised supplementation without formal adherence estimates to self-reported compliance measures.

Importantly, several studies primarily assessed circulating biomarkers of inflammation or bone turnover rather than clinically meaningful or tissue-level outcomes. Thus, changes in markers such as CTX-I, P1NP, osteocalcin, or inflammatory cytokines should not necessarily be interpreted as evidence of improved skeletal health, reduced injury risk, or enhanced long-term recovery. Similarly, short-term post-exercise responses cannot establish sustained effects on muscle hypertrophy, strength development, or sport-specific performance. Overall, the adolescent evidence base remains limited and heterogeneous, with methodological quality ranging from low/poor to moderate-high across studies (Table 2). Current findings provide preliminary support for selected recovery, inflammatory, and bone-remodeling responses, but they do not establish consistent benefits for strength, hypertrophy, body composition, or sport-specific performance. Larger, adequately powered, longer-duration randomized controlled trials with rigorous allocation concealment and blinding where feasible, standardized nutritional assessment, objective adherence monitoring, consideration of biological maturation and sex, systematic adverse-event reporting, and clinically meaningful outcomes are needed to determine the efficacy, safety, and developmental relevance of whey protein supplementation in adolescent athletes.

## 5 Creatine supplementation and high-intensity performance

Creatine supplementation represents a potentially useful nutritional strategy for activities characterized by repeated high-intensity efforts and substantial reliance on the phosphagen energy system. By increasing intramuscular creatine availability, supplementation may increase phosphocreatine availability and support ATP resynthesis during brief, high-power exercise. However, the magnitude and consistency of these effects in adolescent athletes appear to depend on the exercise modality, training status, supplementation protocol, and individual characteristics (7, 60, 61).

### 5.1 PCr system and energy buffering

The PCr system is integral to energy buffering during high-intensity, short-duration physical exertion by facilitating the rapid regeneration of ATP via the creatine kinase reaction. Creatine supplementation can increase intramuscular creatine and PCr availability, providing a larger substrate pool for rapid ATP resynthesis during repeated contractions. This mechanism provides a plausible explanation for improvements observed in some repeated-sprint, jumping, and power-based tasks (28, 62, 63). Nevertheless, increased PCr availability does not necessarily translate into improved performance across all exercise modalities, particularly when performance is strongly determined by aerobic metabolism, technical skill, pacing, or other sport-specific factors.

### 5.2 Rehabilitation and recovery applications

Creatine supplementation has garnered significant scholarly attention for its potential role in rehabilitation and recovery, particularly in conditions involving muscular damage or disuse. In adolescent athletes, evidence for rehabilitation-related applications remains limited but suggests that creatine may help preserve or restore selected aspects of muscle function during periods of injury-related disuse. In an injured adolescent swimmer population, creatine supplementation during rehabilitation was associated with less loss of segmental lean mass, greater recovery of plantar-flexor torque, and lower pain and creatine kinase responses compared with placebo (64–66). However, these findings come from a small clinical study and should not be interpreted as evidence that creatine universally accelerates tissue repair or return to sport. Evidence from adult and experimental models has suggested possible effects on satellite-cell activity, anabolic signaling, and muscle remodeling (67, 68), but these mechanisms have not been sufficiently established in adolescent athletes.

### 5.3 Evidence from clinical trials in adolescents

Collectively, clinical trials conducted in adolescent athletes indicate that creatine supplementation may improve selected performance and rehabilitation-related outcomes, although these effects are highly context-dependent and appear to be influenced by training modality, sport-specific demands, maturational status, and supplementation protocol (Table 3). Rather than producing uniform ergogenic effects across all performance domains, the available evidence suggests greater potential benefit in activities involving repeated high-intensity efforts, explosive muscle actions, and substantial phosphagen-system contribution.

A prominent mechanistic pattern emerging from the available literature is that creatine may support performance through increased phosphocreatine availability and ATP resynthesis, with the clearest benefits reported in repeated sprint performance, jumping ability, muscular power, and sport-specific skills requiring intermittent bursts of maximal effort (69–73). Soccer players receiving creatine supplementation exhibited improvements in dribbling performance, sprint-power tasks, and vertical jump capacity, whereas junior fin swimmers demonstrated enhanced anaerobic power and faster repeated swim times following short-term supplementation (70, 73). Likewise, short-term creatine supplementation combined with resistance training in junior female wrestlers resulted in significant improvements in muscular strength, agility, power, and indices of muscle hypertrophy, suggesting that creatine may augment selected training-related neuromuscular adaptations during adolescence (74). Similarly, when combined with strength and plyometric training, creatine promoted improvements in basketball-specific performance and lower-limb power, suggesting that supplementation may potentiate training-induced neuromuscular adaptations rather than independently increasing performance (72).

In contrast, the evidence is less consistent for outcomes predominantly dependent on aerobic metabolism or technical efficiency. Several investigations reported no significant improvements in endurance performance, isolated sprint outcomes, or overall competitive swimming performance despite favorable changes in physiological variables (75–78). These findings suggest that creatine-induced increases in phosphocreatine availability do not necessarily translate into meaningful improvements in tasks where aerobic energy provision, technical proficiency, pacing strategy, or hydrodynamic efficiency are major determinants of success. Indeed, improvements in laboratory measures of muscular power do not necessarily correspond to enhanced sport-specific performance in highly trained athletes, particularly when baseline performance levels are already elevated.

Another important observation is the potential role of creatine in recovery and rehabilitation. In injured adolescent fin swimmers, creatine supplementation was associated with the restoration of segmental lean mass, improved plantar-flexor strength recovery, attenuated creatine kinase responses, and reduced pain perception during rehabilitation from tendon overuse injuries (79). These findings suggest a possible role for creatine in preserving muscle-related outcomes during rehabilitation, although the small sample size and specific clinical context limit generalization. Emerging evidence from adult populations further suggests that creatine may modulate satellite cell activity, stimulate anabolic signaling pathways such as IGF-1/protein kinase B (Akt)/mTOR, and reduce secondary muscle damage (67, 68). Although these mechanisms have not been directly investigated in adolescents, they provide potential biological explanations rather than confirmed mechanisms for the functional responses observed in youth athletes.

Substantial heterogeneity across studies should, however, be acknowledged. Intervention duration varied considerably, ranging from acute loading protocols lasting 5–14 days to chronic interventions extending up to 8 weeks (69, 70, 72). Supplementation strategies also differed substantially with respect to dosage, loading procedures, and timing of ingestion. Furthermore, participant characteristics varied widely, including differences in sex, age, biological maturation, training status, and competitive level, all of which may influence responsiveness to creatine supplementation. Since adolescence is characterized by profound endocrine and neuromuscular adaptations, maturational differences may partially explain the interindividual variability observed among studies. Importantly, most available trials have predominantly enrolled male athletes, thereby limiting conclusions regarding female adolescent populations. Methodological considerations further contribute to inconsistent findings. Many investigations included relatively small sample sizes (*n* = 4–28), potentially limiting statistical power and increasing the risk of type II error. In addition, outcome measures were highly diverse, encompassing laboratory-based anaerobic tests, sport-specific skills, rehabilitation indices, and competitive performance measures, thereby complicating direct comparisons across studies. The short duration of most interventions also precludes conclusions regarding long-term efficacy and developmental effects.

From a translational standpoint, the current body of evidence suggests that creatine supplementation may be particularly relevant for adolescent athletes participating in intermittent high-intensity sports such as soccer, basketball, wrestling, and sprint swimming, especially during periods of intensified training or rehabilitation (71–73, 79). However, practitioners should recognize that performance benefits are unlikely to be universal and should consider sport-specific energetic demands, training objectives, nutritional status, and biological maturation when implementing creatine supplementation programs. Given the limited duration and small sample sizes of most adolescent trials, these findings support cautious, individualized consideration rather than broad performance claims. Future studies incorporating mechanistic biomarkers, sex-specific analyses, and long-term follow-up are required to refine evidence-based recommendations for creatine use during adolescence.

### 5.4 Critical appraisal of creatine supplementation trials in adolescent athletes: evidence strength and methodological limitations

Across the available trials, the strength of evidence supporting creatine supplementation in adolescent athletes is constrained by substantial methodological heterogeneity and generally small sample sizes. As summarized in Table 4, most studies included relatively small cohorts, frequently with fewer than 30 participants, which limits statistical power and the precision of effect estimates. Moreover, the evidence base is predominantly derived from adolescent or young male athletes, with relatively limited representation of female athletes and mixed-sex populations. This demographic imbalance restricts the generalizability of the findings across sex, developmental stage, and sporting context. Study duration was also variable but generally short, ranging from approximately 1–2 weeks in several supplementation protocols to 6–8 weeks in longer interventions. Consequently, although short-term effects on performance, muscle-related outcomes, and rehabilitation may be detected, the available evidence remains insufficient to establish the long-term efficacy, safety, and developmental implications of creatine supplementation during adolescence. This limitation is particularly relevant because adolescent athletes are undergoing substantial physiological maturation, which may independently influence body composition, strength, power, and performance trajectories.

Methodological quality varied considerably across studies. Several trials incorporated randomized, double-blind, placebo-controlled designs, objective performance assessments, and standardized training protocols, thereby providing relatively stronger evidence. In contrast, other investigations had important limitations, including unclear randomization or allocation-concealment procedures, absence of placebo or blinding, purposive recruitment, small intervention groups, incomplete reporting of adherence, and limited dietary control (Table 4). In some studies, adherence was monitored only through self-report or personal communication rather than through objectively verified measures. Furthermore, differences in creatine dosing strategies, loading procedures, supplementation duration, concurrent training programs, and outcome measures make direct comparison across trials difficult.

Potential confounding by training exposure is another important consideration. Adolescent athletes commonly undergo substantial changes in training volume and intensity, particularly during pre-season or structured rehabilitation programs. Improvements in strength, power, body composition, or sport-specific performance may therefore reflect the combined effects of maturation, training adaptation, and creatine supplementation rather than the supplementation effect alone. Although several studies attempted to standardize or monitor training loads, this was not consistently implemented across investigations. Similarly, dietary intake was not uniformly controlled or quantitatively monitored, despite the potential influence of energy, carbohydrate, protein, and habitual creatine intake on performance and adaptation.

The interpretation of statistical findings also requires caution. Some studies reported significant within-group improvements without significant group × time interactions, whereas others demonstrated significant effects for selected outcomes but not for broader performance measures. Thus, statistical significance should not necessarily be interpreted as evidence of a consistent ergogenic effect of creatine across all performance domains. Small samples, multiple outcome comparisons, and variable analytical approaches further increase uncertainty regarding the magnitude and reproducibility of individual findings. The possibility of performance and expectancy bias cannot be completely excluded, particularly in studies without placebo controls or blinding.

Overall, the evidence suggests that creatine supplementation may provide beneficial effects for selected physical performance and rehabilitation-related outcomes in adolescent athletes, particularly when combined with structured resistance or sport-specific training. However, the certainty and generalizability of these effects remain limited by small samples, short follow-up periods, heterogeneous protocols, incomplete adherence and dietary monitoring, and inconsistent methodological rigor. Therefore, the current literature should be interpreted as promising but not definitive. Future research should prioritize adequately powered, multicenter randomized controlled trials with rigorous allocation procedures, placebo control and blinding where feasible, objectively verified supplementation adherence, standardized dietary and training monitoring, sex-balanced recruitment, and longer follow-up periods to clarify both the efficacy and developmental safety of creatine supplementation during adolescence.

### 5.5 Adolescent-specific safety considerations and long-term uncertainties

Safety considerations warrant particular attention when creatine and protein supplementation are considered for adolescents, given that this population is undergoing rapid growth, skeletal maturation, and physiological development. Although studies involving adolescent athletes have generally reported few adverse events and have not demonstrated consistent evidence of clinically meaningful renal or hepatic dysfunction, the adolescent evidence base remains limited, and most trials were not specifically designed or powered to evaluate long-term safety. A review focused specifically on pediatric and adolescent athletes identified a relatively small number of eligible studies and emphasized the lack of adequately designed long-term safety investigations (60, 80). Therefore, the favorable safety profile of creatine established predominantly in healthy adults should not be interpreted as definitive evidence of long-term safety throughout adolescence. Current pediatric and sports-nutrition guidance supports the potential use of creatine in selected adolescent athletes under appropriate supervision, while emphasizing adherence to recommended doses and appropriate dietary practices (60).

Renal safety warrants particular consideration. Creatine supplementation may increase circulating serum creatinine because creatinine is a normal metabolic product of creatine metabolism; therefore, an isolated increase in serum creatinine does not necessarily indicate impaired kidney function. Available evidence in healthy populations generally does not demonstrate clinically meaningful reductions in glomerular filtration or other consistent markers of renal dysfunction with recommended creatine use (60). Nevertheless, the long-term renal consequences of supplementation specifically during adolescence remain insufficiently characterized. This uncertainty is particularly relevant for adolescents with pre-existing renal disease, recurrent dehydration, concurrent use of potentially nephrotoxic medications, or other factors that may compromise renal function. Importantly, a recent pediatric case report described acute kidney injury with cast nephropathy in a 17-year-old male following a 6-day high-dose creatine loading regimen, despite adequate hydration and the absence of rhabdomyolysis (81). This isolated case does not establish causality or imply that recommended creatine use is generally nephrotoxic, but it underscores the importance of avoiding unsupervised high-dose loading and of considering renal monitoring in higher-risk adolescents. Accordingly, creatine supplementation in adolescents should be approached conservatively and, when considered, should preferably involve appropriate professional supervision.

Hepatic safety has been investigated less extensively in adolescents than in adults. Available short-term studies have not demonstrated consistent increases in conventional markers of hepatic dysfunction; however, small sample sizes and brief intervention periods substantially limit conclusions regarding prolonged exposure during growth. Accordingly, claims of long-term hepatic safety should remain cautious, particularly in adolescents with pre-existing liver disease or those using multiple dietary supplements concurrently. Continued monitoring in future studies should include standardized hepatic biomarkers and systematic reporting of adverse events.

Potential effects on skeletal maturation and growth-plate physiology also warrant consideration. Current adolescent studies have not provided evidence of adverse effects of creatine supplementation on bone maturation or growth-plate development; however, these outcomes have rarely been primary endpoints, and the relatively short duration of available trials limits the ability to exclude subtle or delayed effects on skeletal development. Thus, the absence of reported adverse effects should be interpreted as an absence of demonstrated harm rather than definitive confirmation of long-term skeletal safety. This distinction is particularly important because adolescence represents a period of rapid bone accretion, growth-plate activity, and skeletal maturation.

Protein supplementation similarly requires an individualized, food-first approach. Protein supplements should primarily be used to help meet dietary requirements when necessary rather than to displace carbohydrate, fat, micronutrient-rich foods, or total dietary energy. In adolescent athletes, protein requirements should be considered in relation to body mass, growth and maturation, training volume, sport type, and habitual dietary intake. Pediatric sports-nutrition literature generally indicates that approximately 1.5 g/kg/day of total protein may be sufficient for many youth athletes when energy intake is adequate, with approximately 0.3 g/kg per meal distributed across the day representing a practical intake pattern rather than a rigid requirement (60, 80). Importantly, higher protein intakes substantially exceeding these amounts have not been consistently shown to provide additional performance or developmental benefits in youth athletes, and evidence regarding very high habitual intakes remains limited (80). These values should be interpreted as practical intake guidance rather than a universally established toxicological upper limit, because a definitive evidence-based tolerable upper intake level for protein specifically in adolescent athletes has not been established. Consequently, indiscriminate use of high-protein supplements should be discouraged, and nutritional strategies should prioritize adequate total energy availability, dietary diversity, and individualized protein intake according to growth and training demands.

Taken together, current evidence supports a cautious and individualized rather than categorical interpretation of supplement safety during adolescence. Creatine and protein supplementation may have practical applications in selected adolescent athletes, particularly when dietary requirements cannot be adequately met through food alone, but decisions should consider biological maturation, total dietary intake, training demands, medical history, hydration status, supplement quality, and the limited evidence concerning long-term renal, hepatic, and skeletal outcomes. Larger prospective studies with systematic monitoring of renal and hepatic biomarkers, growth velocity, pubertal maturation, bone development, dietary intake, supplementation adherence, and adverse events are needed before definitive conclusions regarding long-term safety during adolescence can be established.

## 6 Immunometabolism and inter-organ communication in adolescence

Adolescence is distinguished by significant physiological transformations characterized by accelerated growth, maturation of the endocrine system, development of the immune system, and considerable metabolic modifications. Throughout this developmental stage, physical activity and nutritional strategies may influence metabolic and immune processes, providing a physiological context in which immunometabolic regulation may be particularly relevant. Immunometabolism elucidates the reciprocal interactions between cellular metabolic pathways and immune responses that jointly govern tissue adaptation, inflammation, recovery, and the maintenance of overall physiological homeostasis (16). Considering the substantial plasticity of adolescent tissues, adaptations in immunometabolism induced by exercise may possess critical implications not only for athletic performance but also for the long-term trajectories of health (Table 5). However, the extent to which supplementation with whey protein or creatine modifies immunometabolic pathways specifically during adolescence remains incompletely characterized, and available evidence should be distinguished from mechanistic findings derived from adult or experimental models.

### 6.1 Exercise, whey protein, and creatine as modulators of immunometabolic homeostasis

Aerobic physical activity induces temporary inflammatory responses that are generally considered critical for tissue remodeling and physiological adaptation. The contraction of skeletal muscle facilitates the release of a range of signaling molecules that modulate immune function, substrate utilization, and recovery mechanisms. In adolescent swimmers, high-intensity interval swimming provoked marked changes in circulating inflammatory mediators, such as IL-6, IL-10, and TNF-α, indicating that strenuous exercise can acutely alter inflammatory signaling during adolescence (57, 82). Nutritional strategies may additionally affect these inflammatory responses. McKinlay et al. revealed that the ingestion of whey protein post-exercise elevated circulating anti-inflammatory IL-10 levels eight hours after high-intensity interval swimming, in contrast to a decline observed in both carbohydrate and placebo cohorts (57). Although whey protein did not exert a significant impact on exercise performance or creatine kinase levels, the enhanced IL-10 response may indicate a transient shift toward an anti-inflammatory recovery response (57). Importantly, this finding should not be interpreted as evidence that whey protein broadly suppresses exercise-induced inflammation, because the observed effect was restricted to a specific biomarker and time point.

In a similar vein, Bell et al. identified modest modifications in inflammatory biomarkers, such as interleukin-1 beta (IL-1β), IL-6, IL-10, and TNF-α, subsequent to chronic protein supplementation in adolescent athletes, suggesting that dietary protein sources may exert influence on immune regulation in a manner that is specific to sex (56). The variability of these responses further suggests that inflammatory effects may depend on protein source, sex, training status, and the broader nutritional context rather than reflecting a uniform immunomodulatory effect.

Furthermore, creatine supplementation may also play a role in the regulation of immunometabolic processes. Apart from its well-documented function in the resynthesis of phosphocreatine, creatine has been reported to attenuate selected markers of oxidative stress and inflammatory responses that occur after repeated sprint exercise (83). Such effects may facilitate improved recovery and the maintenance of cellular homeostasis during periods of heightened training intensity. Nevertheless, evidence for these immunometabolic effects is substantially less developed in adolescents than evidence concerning creatine’s role in phosphagen energy metabolism.

Overall, the available evidence supports a model in which exercise provides the primary physiological stimulus, while whey protein and creatine may modify selected components of the recovery and metabolic response. However, direct evidence demonstrating a synergistic interaction among these interventions in adolescent populations remains limited.

### 6.2 Myokines, osteokines, adipokines, and inflammatory mediators

Skeletal muscle is increasingly acknowledged as an endocrine organ that possesses the capability to secrete a variety of cytokines and peptide factors, collectively referred to as myokines, in reaction to muscular contraction (17). These biomolecules exert autocrine, paracrine, and endocrine effects, thereby facilitating intercellular communication between skeletal muscle and remote organs, including osseous tissue, adipose tissue, hepatic tissue, and the central nervous system. Among the exercise-responsive mediators that have been extensively examined, IL-6 stands out. Although it has been traditionally classified as a pro-inflammatory cytokine, exercise-induced IL-6 can act as a metabolic modulator by enhancing glucose uptake, promoting lipid oxidation, and initiating anti-inflammatory pathways (17). Research involving adolescent swimmers has documented acute elevations in IL-6 levels following strenuous swimming sessions, thereby supporting its involvement in the acute immunometabolic response to physical activity (57, 82).

Furthermore, exercise-induced increases in IL-10 may contribute to the regulation of inflammatory responses by counteracting pro-inflammatory signaling. Whey protein supplementation has been shown to increase circulating IL-10 concentrations following high-intensity interval swimming in adolescent athletes, which may indicate an acute anti-inflammatory response during recovery (57). Conversely, TNF-α and IL-1β may exhibit more heterogeneous responses depending on exercise and nutritional conditions, with some responses showing sex-specific patterns in adolescent athletes (56).

In addition to established cytokines, inter-organ communication may also involve signaling molecules including irisin, IGF-1, osteocalcin, and osteoprotegerin (OPG). Seasonal training regimens in adolescent swimmers have been correlated with synchronized alterations in IL-6, IL-10, TNF-α, and circulating concentrations of IGF-1, suggesting a possible association between inflammatory and anabolic signaling pathways (82). Furthermore, protein supplementation may influence selected bone-derived mediators and markers of bone remodeling, including OPG, CTX-I and sclerostin, with some responses showing sex-specific patterns in adolescent athletes (56). These findings provide evidence for potential interactions between nutritional protein intake and muscle–bone signaling during periods of growth and training, although the observed responses were modest and variable.

Recent mechanistic investigations further indicate that exercise-induced myokines, particularly irisin, have the potential to enhance BDNF expression via the PGC-1α/FNDC5 signaling pathway, thereby establishing a mechanistic connection between skeletal muscle activity and neuroplasticity (18). However, this pathway should currently be regarded primarily as a mechanistic framework rather than a confirmed whey protein– or creatine-mediated pathway in adolescents, because direct intervention studies linking supplementation, circulating irisin, and downstream BDNF signaling in this population remain limited.

Collectively, these findings support the concept of muscle–bone–immune–brain communication during adolescent exercise, but they also highlight the distinction between observed biomarker associations and established causal inter-organ signaling mechanisms.

### 6.3 Redox signaling and oxidative stress regulation

Reactive oxygen species (ROS) produced during physical exertion were historically regarded exclusively as harmful metabolic by-products. Nevertheless, recent research suggests that moderate elevations in ROS function as critical signaling molecules that regulate mitochondrial biogenesis, antioxidant defense mechanisms, and cellular adaptation processes (84). Consequently, exercise-induced redox signaling constitutes a pivotal aspect of physiological adaptation. Adolescents engaged in consistent aerobic training display an augmented endogenous antioxidant capacity and enhanced resistance to oxidative stress when juxtaposed with their sedentary counterparts (38). However, excessive training regimens may perturb redox homeostasis, potentially contributing to muscle damage, inflammation, and compromised recovery.

Creatine supplementation has been identified as a plausible modulator of oxidative stress. Deminice et al. illustrated that the administration of creatine resulted in a reduction of biomarkers associated with oxidative stress following repeated sprint exercise, thereby proposing a protective role against cellular damage induced by exercise (83). Nevertheless, these findings provide evidence for modulation of selected oxidative-stress markers rather than definitive proof that creatine prevents exercise-induced cellular damage, particularly in adolescent populations.

Similarly, whey protein may play an indirect role in bolstering antioxidant defenses by supplying cysteine, a precursor necessary for the biosynthesis of glutathione, one of the primary endogenous antioxidants within the human body (52). This mechanism is biologically plausible; however, the availability of cysteine from whey protein should not be equated with a demonstrated increase in glutathione-dependent antioxidant capacity in adolescent athletes unless directly measured.

At the molecular level, redox-sensitive transcription factors, including nuclear factor erythroid 2-related factor 2 (Nrf2), nuclear factor-kappa B (NF-κB), and PGC-1α, orchestrate the cellular responses to oxidative stress and facilitate mitochondrial adaptations (85).

Accordingly, exercise, whey protein, and creatine may converge on partially overlapping metabolic and redox-regulatory processes, but the available adolescent evidence does not yet establish that their combined use produces additive or synergistic effects on redox homeostasis. A more cautious interpretation is that exercise provides the principal adaptive stimulus, whereas nutritional supplementation may influence selected components of energy metabolism, recovery, antioxidant defense, or inflammatory signaling depending on the physiological and nutritional context.

Taken together, the current evidence supports an emerging muscle–immune–bone–brain communication framework in adolescence, but substantial mechanistic and clinical uncertainties remain. In particular, future studies should determine whether changes in circulating cytokines and other inter-organ mediators translate into meaningful functional adaptations and whether these responses are modified by sex, biological maturation, training load, baseline nutritional status, and supplementation strategy.

## 7 Muscle–bone–brain crosstalk and neuroplasticity

The notion of muscle–bone–brain crosstalk has emerged as a framework within exercise physiology for understanding endocrine and metabolic interactions among skeletal muscle, bone, adipose tissue, and the central nervous system. Throughout adolescence, a developmental stage characterized by accelerated somatic growth, skeletal maturation, and neurodevelopment, this inter-organ communication may contribute to physiological adaptation to exercise and nutritional interventions. Skeletal muscle contraction promotes the release of bioactive molecules, including myokines and metabolites, which can interact with bone-derived factors and neurotrophic mediators to influence metabolism, tissue remodeling, and brain function (17) (Figure 3 and Table 6). Importantly, the evidence supporting these pathways comes from different experimental populations and models. Some mechanisms have been demonstrated in adolescent athletes, whereas others are based on animal models, cell cultures, or adult and older-adult populations. Therefore, the mechanistic pathways described below should be interpreted as biological plausibility rather than as evidence that the same effects have been directly established in adolescents. This distinction is particularly important when discussing neuroplasticity and muscle–bone–brain crosstalk during adolescence.

**FIGURE 3:**
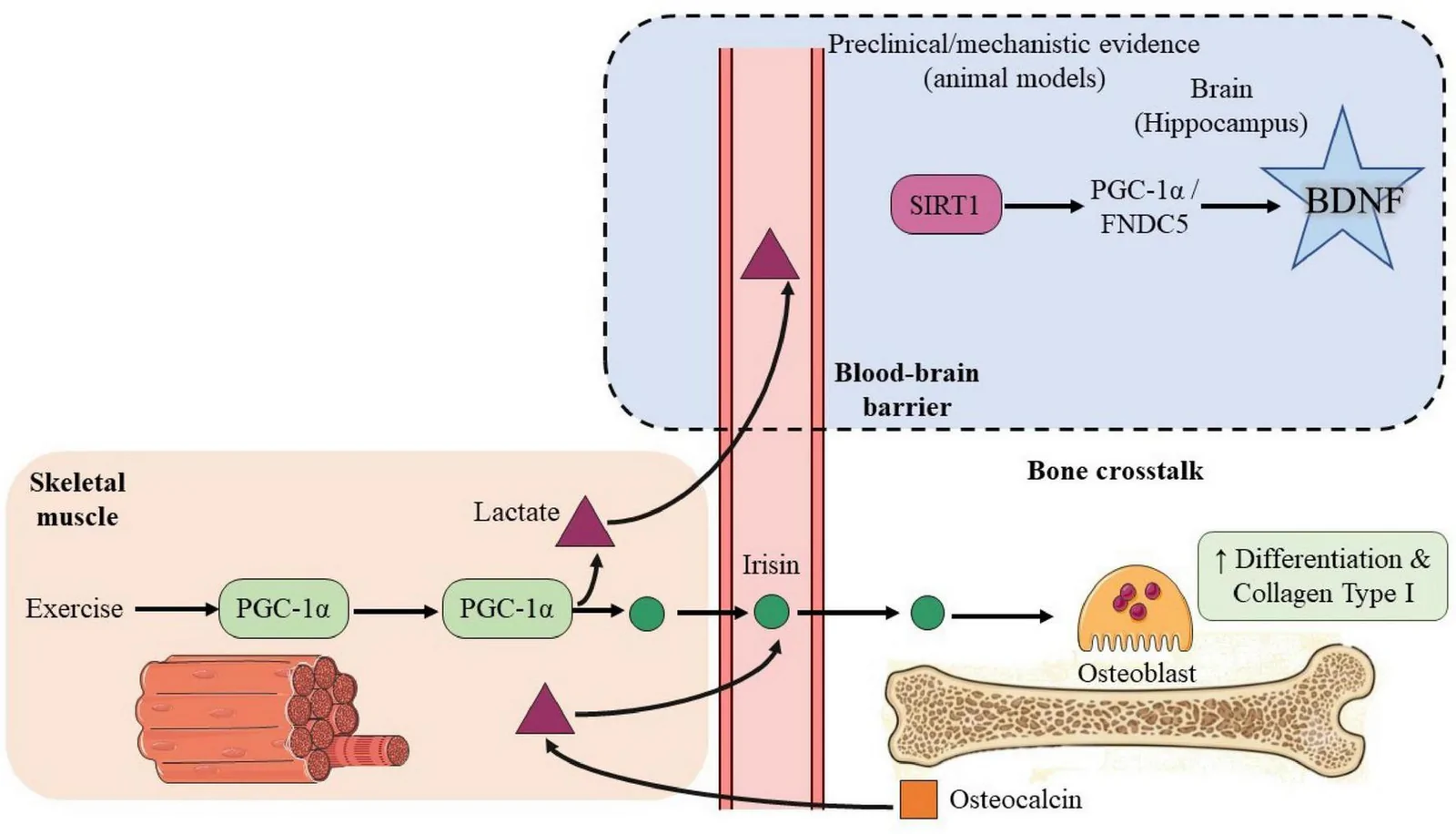
Mechanistic pathways of exercise-induced muscle–bone–brain crosstalk. This figure outlines the systemic endocrine function of skeletal muscle during exercise, specifically the secretion of lactate and irisin (via the PGC-1α/FNDC5 pathway). Circulating irisin forms a bidirectional feedback loop with bone by stimulating osteoblast differentiation and the subsequent release of osteocalcin. The neurological and osteogenic pathways depicted, specifically the lactate- and irisin-mediated upregulation of hippocampal BDNF and osteoblast differentiation, are predominantly based on preclinical animal models (indicated by the dashed boundary) and require further validation in adolescent human cohorts.

### 7.1 Skeletal muscle as an endocrine organ: myokine signaling

One of the most studied pathways linking skeletal muscle and brain function is the PGC-1α/FNDC5/BDNF signaling axis. Exercise-induced activation of PGC-1α in skeletal muscle promotes FNDC5 expression, which can generate irisin (86). Boström et al. reported that exercise increased PGC-1α and FNDC5 expression in skeletal muscle and increased circulating irisin, together with greater energy expenditure and browning of white adipose tissue (86). These findings were derived from experimental models and human exercise studies and mainly support the role of the PGC-1α/FNDC5/irisin pathway in metabolic adaptation; they do not establish the same mechanism specifically in adolescents.

Beyond its metabolic effects, the FNDC5/irisin pathway has also been linked to brain function. Wrann et al. showed in mouse models and neuronal experiments that prolonged endurance exercise increased FNDC5 expression in the hippocampus, whereas FNDC5 overexpression increased BDNF expression and neuroprotective gene transcription (18). Conversely, reduced FNDC5 expression was associated with lower BDNF expression in these experimental models (18). These findings provide preclinical evidence for a possible FNDC5/BDNF pathway linking exercise-related signaling with neuroplasticity, but they should not be interpreted as direct evidence in adolescent humans.

BDNF is an important neurotrophin involved in neuronal survival, synaptic plasticity, learning, and memory (87). Because adolescence is a period of active brain development, the PGC-1α/FNDC5/BDNF pathway is biologically relevant to this population. However, direct evidence demonstrating that aerobic exercise, whey protein, or creatine activates this complete pathway and produces corresponding neuroplastic adaptations in adolescents remains limited.

### 7.2 Exercise-derived metabolites and muscle–brain communication

In addition to myokines, physical exercise produces metabolites that can act as signaling molecules. Lactate, traditionally considered a metabolic by-product, is now recognized as a potential mediator of communication between skeletal muscle and the brain (88).

El Hayek et al. reported in a male mouse model that exercise-derived lactate crossed the blood–brain barrier and increased hippocampal BDNF expression through a sirtuin 1 (SIRT1)-dependent pathway (86). Specifically, lactate increased SIRT1 activity, which was followed by increased PGC-1α/FNDC5 signaling and BDNF expression, together with improved spatial learning and memory performance (89). These findings provide mechanistic evidence that lactate may link exercise-related metabolism with brain plasticity. However, because the study was conducted in mice, the lactate/SIRT1/PGC-1α/FNDC5/BDNF pathway should be considered a preclinical mechanism rather than a pathway directly demonstrated in adolescents. Human adolescent studies are needed to determine whether similar metabolic signaling occurs during aerobic exercise and whether it is associated with measurable changes in cognition or motor learning.

### 7.3 Bone-derived factors, neurotrophins, and muscle–bone communication

Skeletal muscle and bone communicate through endocrine and paracrine factors that can influence tissue growth and remodeling (19). Irisin has emerged as a potential mediator of this interaction. Colaianni et al. demonstrated in mouse-derived cells and exercised mice that irisin increased osteoblast differentiation and the expression of osteogenic markers, including alkaline phosphatase and collagen type I (90). These findings provide preclinical evidence for irisin-mediated muscle-to-bone signaling, but direct evidence that this mechanism occurs in adolescents is currently lacking.

Bone also functions as an endocrine organ by releasing osteokines that may influence muscle metabolism and function. Osteocalcin, particularly its undercarboxylated form (ucOC), has received attention because of its potential role in musculoskeletal function and energy metabolism (91). In a *post-hoc* analysis of women older than 70 years, relative ucOC was positively associated with muscle strength, whereas lower ucOC was associated with higher concentrations of the inflammatory marker alpha-actin (92). Because these findings were obtained from an older-adult population, they cannot be directly extrapolated to adolescents. Nevertheless, they provide biological plausibility for bone-derived endocrine signaling and highlight osteocalcin as a potential component of muscle–bone communication. Whether similar associations occur during the rapid bone accretion and muscle development of adolescence remains unclear.

Evidence directly obtained from adolescents provides a more relevant basis for evaluating nutrition-related muscle–bone and inflammatory interactions. McKinlay et al. reported that adolescent swimmers receiving post-exercise whey protein following high-intensity interval swimming showed higher IL-10 concentrations during recovery, whereas IL-6, creatine kinase, and performance outcomes did not differ significantly between groups (57). These findings provide direct adolescent evidence that whey protein may selectively influence the acute inflammatory response during post-exercise recovery; however, they do not demonstrate a direct effect on the complete muscle–bone–brain crosstalk network.

Similarly, Bell et al. investigated Greek yogurt versus whey protein supplementation during a competitive season in adolescent athletes and reported modest and frequently sex-specific changes in bone and inflammatory biomarkers (56). Changes were observed in osteocalcin, OPG, CTX-I, and sclerostin, while IL-1β, IL-6, IL-10, and TNF-α also showed variable responses (56). Thus, this study provides direct adolescent evidence that protein intake may influence selected bone-remodeling and inflammatory markers, but the findings do not establish a uniform anabolic or anti-inflammatory effect.

### 7.4 Integrated interpretation of muscle–bone–brain signaling

In aggregate, the available evidence suggests that exercise and nutritional factors may influence a network of signaling molecules, including irisin, lactate, BDNF, osteocalcin, OPG, and inflammatory cytokines, that contribute to communication among skeletal muscle, bone, and brain. However, the strength of evidence differs substantially across these pathways. Evidence from adolescent athletes is currently stronger for selected inflammatory and bone-related biomarkers than for direct measures of neuroplasticity or complete muscle–bone–brain signaling. Preclinical studies provide important mechanistic evidence for the PGC-1α/FNDC5/irisin/BDNF and lactate/SIRT1/PGC-1α/FNDC5/BDNF pathways, whereas adolescent studies provide more direct evidence for selected responses to whey protein and exercise. Therefore, these pathways should be viewed as an integrated mechanistic framework that generates biologically plausible hypotheses rather than as a fully established adolescent-specific pathway. This distinction is particularly important when considering cognitive adaptation, motor learning, and long-term neurodevelopment. Although BDNF and related signaling pathways are biologically relevant to these processes, the adolescent studies summarized in this review have not directly established that whey protein or creatine, alone or combined with aerobic exercise, produces changes in validated measures of neuroplasticity, motor learning, or physical literacy. Further adolescent-specific studies that simultaneously assess exercise exposure, nutritional intake, circulating signaling molecules, and functional neurocognitive or motor outcomes are therefore needed. Overall, the muscle–bone–brain crosstalk framework provides a useful model for understanding how exercise and nutritional factors may interact during adolescence, while the current evidence supports cautious interpretation of mechanistic findings derived from animal and adult studies.

## 8 Molecular and cellular mechanisms underlying exercise–nutrition synergy

Adolescence constitutes a pivotal period for neuromuscular plasticity, wherein metabolic and mechanical stimuli converge to influence long-term functional capacity. Engaging in aerobic exercise promotes mitochondrial biogenesis and oxidative metabolism, while dietary interventions such as whey protein and creatine supplementation may influence anabolic and energetic pathways. At the molecular level, physical activity triggers essential signaling pathways including AMPK, mTOR, and PGC-1α, thereby integrating energy sensing with muscle remodeling processes. Whey protein supplies essential amino acids, with a particular emphasis on leucine, which can stimulate anabolic signaling, while creatine augments phosphocreatine availability, thereby supporting rapid ATP resynthesis during successive muscle contractions. These physiological adaptations may contribute to adaptations in muscle fiber characteristics, motor unit recruitment, and intercellular communication mediated by myokines. Although these pathways provide a plausible biological basis for interactions between exercise and nutritional interventions, direct evidence demonstrating synergistic effects of combined whey protein and creatine supplementation on metabolic flexibility or neuromuscular coordination in adolescents remains limited. A comprehensive understanding of these multi-faceted mechanisms offers valuable insights into the potential integration of training and nutritional strategies throughout the growth phase (5, 14, 93).

### 8.1 MPS and anabolic signaling

MPS serves as a pivotal factor influencing muscular adaptation during the adolescent period, governed by the equilibrium between anabolic and catabolic signaling pathways. Aerobic exercise, while conventionally linked to endurance enhancement, may influence protein turnover and anabolic signaling through changes in nutrient delivery, energy metabolism, and insulin sensitivity. The consumption of whey protein leads to a rapid increase in plasma amino acid concentrations, especially leucine, which in turn can stimulate the mTORC1 signaling pathway, thereby promoting translation initiation and supporting translational activity. Furthermore, the supplementation of creatine may influence muscle adaptation through effects on cellular energy availability and, based largely on mechanistic and adult evidence, satellite-cell-related processes. Additionally, the interaction between AMPK and mTOR pathways provides a mechanism through which cellular energy status can interact with the demands of protein synthesis. In the adolescent population, these physiological mechanisms may occur within a developmental environment characterized by substantial anabolic signaling and changes in growth hormone and IGF-1 activity. Consequently, the combined provision of exercise stimuli and adequate protein and energy availability may support anabolic processes and muscle adaptation; however, the extent to which creatine provides an additional anabolic effect in healthy adolescents remains incompletely established (6, 94, 95).

### 8.2 Inflammatory modulation and recovery pathways

Exercise-induced inflammation represents a tightly regulated phenomenon that is critical for muscle repair and adaptation. In the adolescent population, moderate aerobic exercise may induce dynamic changes in inflammatory mediators, including TNF-α, IL-6, and IL-10, with the magnitude and direction of these responses depending on exercise intensity, duration, training status, and the biomarker assessed. The incorporation of whey protein into recovery protocols facilitates the provision of cysteine, which serves as a precursor for glutathione biosynthesis, thus potentially supporting glutathione synthesis and antioxidant defenses. Additionally, creatine supplementation may influence recovery-related processes through effects on cellular energy availability and, potentially, markers of exercise-induced muscle damage, including creatine kinase. On a molecular scale, these interventions may interact with NF-κB and other redox-sensitive signaling pathways involved in inflammatory regulation, tissue remodeling, and recovery. Collectively, these mechanisms provide a plausible basis for interactions between exercise and nutritional strategies in regulating inflammatory and recovery-related processes during adolescence. However, direct evidence demonstrating a synergistic effect of exercise combined with whey protein and creatine on inflammatory resolution or athletic performance in adolescents remains limited. Therefore, these pathways should be interpreted as mechanistically plausible rather than as established synergistic effects (96–98).

### 8.3 Neuromuscular efficiency and fatigue resistance

Neuromuscular efficiency and resistance to fatigue constitute pivotal factors influencing motor performance in the adolescent population. Engagement in aerobic exercise promotes adaptations in mitochondrial oxidative capacity and, over time, vascular and metabolic support for oxygen delivery and utilization. The administration of creatine supplementation elevates phosphocreatine reserves, thereby facilitating the swift resynthesis of ATP during high-intensity or repetitive physical activities. This mechanism provides a plausible explanation for the greater relevance of creatine to repeated high-intensity efforts than to predominantly aerobic performance. Whey protein contributes to the provision of amino acids required for muscle protein turnover and remodeling, although direct evidence linking whey supplementation to improved neuromuscular efficiency in adolescents remains limited.

Changes in motor-unit recruitment, firing frequency, and synchronization may contribute to neuromuscular adaptation to training, but these neural mechanisms have not been consistently demonstrated as direct effects of whey protein or creatine supplementation in adolescents. Similarly, claims that creatine or whey protein reduces lactate or hydrogen-ion accumulation should be interpreted cautiously unless these variables are directly measured. Accordingly, the available evidence supports a potential contribution of creatine to repeated high-intensity energy provision, whereas the independent effects of whey protein on neuromuscular efficiency and fatigue resistance remain less clearly established (28, 60, 99).

### 8.4 mTOR–AMPK–PGC-1α signaling network

The mTOR, AMPK, and PGC-1α form a cohesive signaling network that orchestrates skeletal muscle adaptation, energy metabolism, and inter-organ communication in response to exercise and nutritional inputs. This network assumes particular significance during adolescence, a developmental stage marked by accelerated growth, heightened metabolic demands, and increased tissue plasticity. mTOR functions as a pivotal nutrient-sensing kinase that facilitates protein synthesis, muscle hypertrophy, and cellular proliferation in response to amino acids, insulin, and mechanical stimuli (100). Whey protein, due to its elevated leucine concentration, can stimulate mTORC1, thereby enhancing translation initiation and fostering muscle protein accumulation (101). Consequently, in adolescent athletes, sufficient protein consumption provides the amino acid substrate required for growth, tissue remodeling, and training adaptation, with mTORC1 representing one important molecular pathway through which these effects may be mediated.

Conversely, AMPK operates as a cellular energy sensor that is activated in response to energetic stressors, such as sustained aerobic exercise. The activation of AMPK promotes glucose uptake, enhances fatty acid oxidation, and stimulates mitochondrial biogenesis, while concurrently suppressing mTOR signaling under conditions characterized by restricted energy availability (41). Notably, AMPK directly phosphorylates and activates PGC-1α, which serves as a major regulator of mitochondrial biogenesis and oxidative metabolism (42). Furthermore, PGC-1α facilitates the secretion of exercise-induced myokines, including FNDC5/irisin, thereby establishing a connection between skeletal muscle activity and adaptations in bone and brain (18). This proposed muscle–brain and muscle–bone communication pathway is biologically plausible, but direct evidence linking whey protein or creatine supplementation to PGC-1α–FNDC5/irisin-mediated neuroplastic or skeletal outcomes in adolescents remains limited.

Through the coordinated modulation of anabolic and oxidative pathways, the mTOR–AMPK–PGC-1α signaling axis provides a conceptual framework for understanding how nutritional availability and exercise-induced energetic stress may converge on muscle adaptation and metabolic regulation during adolescence. The dysregulation of this intricate network may be associated with compromised recovery, impaired mitochondrial function, and hindered long-term physiological adaptation (14).

### 8.5 Mitochondrial adaptations and cellular bioenergetics

Mitochondria function as pivotal modulators of cellular bioenergetics, orchestrating substrate utilization, ATP synthesis, redox homeostasis, and intracellular signaling pathways. The engagement in aerobic exercise prompts adaptive mitochondrial modifications that augment oxidative capacity and metabolic flexibility, phenomena that hold particular relevance during the developmental stage of adolescence due to heightened energetic requirements concomitant with growth and maturation. The repetitive practice of aerobic exercise incites mitochondrial biogenesis predominantly through the activation of PGC-1α, which governs the transcriptional regulation of both nuclear and mitochondrial genes implicated in oxidative phosphorylation (44). Upstream modulators, including AMPK and p38 mitogen-activated protein kinase, respond to exercise-induced energetic stressors and facilitate the activation of PGC-1α, thereby contributing to mitochondrial remodeling and oxidative capacity (14). These physiological adaptations may support endurance capacity, metabolic flexibility, and fatigue resistance.

Exercise-induced mitochondrial remodeling is concomitantly associated with augmented antioxidant defenses. Moderate elevations in ROS generated during physical activity function as signaling molecules that stimulate mitochondrial biogenesis and activate endogenous antioxidant mechanisms via redox-sensitive pathways, including Nrf2 (84). Nevertheless, excessive oxidative stress has the potential to compromise mitochondrial integrity and impede recovery processes. Nutritional interventions may interact with these processes. Whey protein delivers essential amino acids and cysteine, thereby bolstering MPS and providing substrates that may support glutathione synthesis and redox regulation (52). Furthermore, creatine supplementation may enhance cellular bioenergetics by increasing the availability of phosphocreatine, thereby facilitating rapid ATP regeneration and potentially attenuating selected markers of exercise-induced oxidative stress (83).

Collectively, these mechanisms suggest that aerobic exercise provides a major stimulus for mitochondrial remodeling, whereas adequate protein availability and creatine supplementation may influence complementary aspects of muscle protein turnover and cellular energy buffering. Nevertheless, direct evidence demonstrating that their combination produces synergistic mitochondrial adaptations in adolescents remains insufficient. Future studies should therefore distinguish between mechanistic plausibility and clinically demonstrated effects, particularly by examining mitochondrial biomarkers, training status, sex, biological maturation, dietary adequacy, and long-term functional outcomes.

## 9 Motor competence-related and functional adaptations

Motor competence constitutes a pivotal element of healthy growth and athletic progression throughout the adolescent phase, encompassing the capability to execute a diverse array of movement skills, including agility, coordination, balance, and sport-specific techniques. This developmental stage is distinguished by significant neurobiological plasticity, rapid advancement of the neuromuscular system, and increased responsiveness to training stimuli, thereby rendering adolescence a crucial period for the enhancement of motor performance and physical literacy (102). Emerging research indicates that the amalgamation of exercise training with focused nutritional interventions, particularly through whey protein and creatine supplementation, may support selected functional adaptations by contributing to energy availability, muscle remodeling, and recovery. However, direct evidence that whey protein or creatine independently enhances overall motor competence in adolescents remains limited (Table 7).

### 9.1 Sport-specific skills and neuromuscular performance

Numerous intervention studies suggest that creatine supplementation may exert a favorable influence on selected motor competence-related outcomes among adolescent athletes. In a cohort of young soccer players, a 7-day regimen of creatine monohydrate supplementation resulted in significant enhancements in dribbling performance, sprint-power performance, and vertical jump height, all while preserving endurance capacity (73). These observations are consistent with the proposed role of increased phosphocreatine availability in supporting rapid ATP resynthesis during explosive and repeated high-intensity activities, although the extent to which this metabolic effect directly improves motor execution may depend on the sport-specific task. Similarly, Juhasz et al. (70) illustrated that a 5-day creatine supplementation regimen in elite junior fin swimmers led to a notable increase in average mechanical power output by approximately 20% and improved repeated maximal swimming performance. Correspondingly, the combination of creatine supplementation with resistance and plyometric training yielded enhancements in Abalakov jump performance and scoring ability among under-16 basketball players, indicating potential benefits for lower-limb power and sport-specific performance (72). Although chronic supplementation with creatine during pre-season training did not yield a statistically significant enhancement in countermovement jump performance among elite soccer athletes, it was associated with attenuation of the decline in lower-limb power that is typically observed during periods of intensive training (78).

Conversely, the body of evidence concerning the effects of whey protein on motor performance is markedly inconsistent. Zimmerman et al. (58) indicated that a 10-week regimen of whey protein supplementation led to improvements in aerobic fitness (V̇O_2_max) among adolescent soccer players, yet it did not result in concomitant enhancements in sprint performance or muscular strength. Although improved aerobic fitness may support the physiological demands of repeated exercise, the absence of corresponding improvements in sprint performance or strength in this study does not allow a direct conclusion that whey protein enhances motor competence.

In addition to nutritional strategies, the act of exercising itself appears to impart advantageous effects on cognitive-motor performance. It was established that acute episodes of endurance, strength, and coordination exercises significantly enhanced attentional performance in adolescents, irrespective of the specific exercise modality employed (103). Considering the intricate relationship among attention, executive function, and the acquisition of motor skills, the results of this investigation provide a plausible basis for considering cognitive processes as one contributor to motor performance, although improvements in attention should not be equated directly with improved motor competence.

In summary, the prevailing body of evidence suggests that creatine may be more consistently relevant to selected high-intensity and explosive performance tasks, whereas evidence for direct effects of whey protein on motor performance is less consistent. Importantly, these findings should be interpreted as task- and sport-specific effects rather than evidence that either supplement broadly improves motor competence during adolescence.

### 9.2 Cognitive and neuromuscular processes relevant to motor learning

Adolescence is marked by significant maturation of both the central and peripheral nervous systems, which encompasses increased myelination, the refinement of corticospinal pathways, and the enhancement of motor unit recruitment strategies (104). As a result, this developmental phase is especially responsive to interventions aimed at improving neuromuscular performance and motor learning. Motor learning is contingent upon repetitive practice and neural adaptations that enhance movement efficiency, coordination, and precision. Integrative neuromuscular training (INT), which amalgamates resistance exercises, dynamic stability tasks, plyometric activities, and skill practice, has been empirically demonstrated to significantly bolster both health-related and skill-related fitness in youth populations. Faigenbaum et al. (105) documented notable enhancements in long jump performance, single-leg hopping capabilities, running performance, and muscular fitness subsequent to an 8-week regimen of INT incorporated into physical education curricula. These results support the potential of structured neuromuscular training to improve selected components of movement and physical fitness during youth.

Emerging research also indicates that nutritional interventions may play a supportive role in neuromuscular development. Creatine supplementation enhances the stores of intramuscular phosphocreatine, consequently augmenting ATP buffering capacity and facilitating repeated high-intensity muscular contractions. The increased availability of energy may promote more effective motor practice and postpone the onset of neuromuscular fatigue, thereby enabling athletes to execute a greater number of high-quality movement repetitions (72, 73). However, whether these metabolic effects translate into superior motor learning or long-term skill acquisition has not been directly established in adolescent populations.

Moreover, creatine may have a direct impact on neural function. Findings from experimental investigations suggest that creatine is involved in brain energy metabolism and may bolster cognitive functions, such as memory retention and information processing (106). Although research concerning adolescents is still limited, the enhancement of neural energy homeostasis could potentially contribute to motor learning and the acquisition of skills. This proposed cognitive–motor effect remains mechanistic and should not be interpreted as evidence that creatine supplementation directly improves motor learning in adolescent athletes.

Whey protein may also play an indirect role in neuromuscular development due to its elevated leucine concentration and its capacity to stimulate MPS. By facilitating muscle repair and remodeling processes, whey protein may augment the structural adaptations requisite for enhancements in movement proficiency and training responsiveness (58). Furthermore, the attenuation of muscle soreness following whey protein consumption after high-intensity interval swimming may support perceived recovery in adolescent athletes, although its implications for subsequent training participation or motor-skill practice remain to be established (57). Thus, the potential contribution of whey protein to motor competence is more plausibly mediated through recovery and muscle remodeling than through a direct effect on neural motor learning.

From a mechanistic standpoint, the exercise-induced activation of the AMPK–PGC-1α signaling pathway and the resultant increases in BDNF may serve as a pivotal nexus linking physical activity with motor learning (18). BDNF is known to promote synaptic plasticity, neuronal survival, and the consolidation of motor memory, thereby providing a biologically plausible mechanism through which exercise may influence neural plasticity relevant to motor learning. Nevertheless, evidence directly demonstrating that whey protein or creatine supplementation modifies the AMPK–PGC-1α–BDNF pathway to improve motor learning in adolescents remains insufficient.

### 9.3 Long-term athletic development and the potential relevance of physical literacy

Motor competence during adolescence constitutes a pivotal foundation for the trajectory of long-term athletic development and enduring engagement in physical activity. Physical literacy, which encompasses the motivation, confidence, physical competence, knowledge, and understanding requisite for sustained participation in physical activity, is increasingly acknowledged as a fundamental factor influencing both athletic achievement and long-term health outcomes (107). However, the adolescent studies identified in the present review did not directly assess physical literacy using validated physical-literacy instruments. Accordingly, the potential relationship between the exercise and nutritional interventions considered here and physical literacy remains inferential rather than directly demonstrated. Adolescents who attain elevated levels of motor competence may be more likely to maintain physical activity, exhibit enhanced performance in sports, and demonstrate a reduced risk of injury. Nevertheless, the outcomes examined in the available adolescent studies including sprint performance, jumping ability, dribbling, repeated-sprint performance, aerobic capacity, and attention, should be interpreted primarily as motor competence-related, neuromuscular, cognitive, or sport-specific performance measures rather than as direct measures of physical literacy (70, 72, 78). Whey protein and creatine may support selected aspects of recovery, training adaptation, and high-intensity performance; however, whether these effects contribute meaningfully to the development or maintenance of motor competence remains uncertain (57, 58). Importantly, these findings do not establish that either supplement directly improves physical literacy. It is crucial to consider the interactions among physical exercise, nutritional factors, and neurobiological adaptations within a comprehensive systems framework. Enhancements in energy metabolism, immunometabolic regulation, mitochondrial functionality, and the communication between muscle and brain may plausibly contribute to the improvement of motor competence and the long-term development of athletic prowess. Similarly, cognitive responses to exercise, such as improvements in attention, may be relevant to processes involved in motor learning and skill acquisition, but should not be interpreted as direct evidence of motor learning in the absence of validated longitudinal skill-acquisition assessments. Thus, the implementation of integrated training and nutritional strategies during the adolescent period may yield lasting benefits for motor competence, sport-specific performance, and physical activity participation; however, whether such strategies directly influence physical literacy or long-term athletic development remains to be established through prospective adolescent-specific studies.

## 10 Limitations and methodological considerations

Several methodological limitations identified across the studies included in this review warrant careful consideration when interpreting the current body of evidence. One of the most prominent limitations is the relatively small sample size employed in many investigations, which reduces statistical power, increases the likelihood of type II error, and limits the detection of subtle but potentially meaningful effects. Furthermore, most interventions were characterized by short durations, ranging from acute supplementation protocols to only a few weeks of training, thereby restricting conclusions regarding long-term physiological adaptations, developmental safety, and sustained performance outcomes during adolescence. Considerable heterogeneity was also observed across studies with respect to participant characteristics, biological maturation status, sex distribution, training experience, sport discipline, exercise modality, supplementation protocol, and outcome assessment. Variations in training interventions, including swimming, soccer, resistance training, plyometric exercise, and mixed training paradigms, complicate direct comparisons among studies and limit the generalizability of findings to broader adolescent athletic populations. This heterogeneity also makes it difficult to determine whether observed responses reflect effects of the supplements themselves, interactions with specific training modalities, or differences in baseline nutritional and training status.

Another important methodological consideration is the limited assessment of biological maturation and pubertal status. Given that adolescence is characterized by profound endocrine, musculoskeletal, and neurodevelopmental changes, failure to account for maturation stage may substantially influence the interpretation of training and supplementation responses. Age alone may therefore be insufficient to capture the biological variability within adolescent cohorts. Similarly, dietary intake, habitual physical activity, sleep quality, and recovery practices were inconsistently monitored across studies, potentially confounding observed outcomes. Although many investigations employed randomized, placebo-controlled, or double-blind designs, several studies inadequately reported adherence rates, effect sizes, allocation procedures, or adverse events, thereby increasing the risk of bias and reducing external validity. In addition, differences in comparator conditions, including carbohydrate, water, placebo, or alternative protein sources, further complicate interpretation because the observed response may depend on what the supplementation replaces rather than on an independent effect of whey protein or creatine. Importantly, substantial gaps remain regarding the mechanistic understanding of exercise–nutrition interactions during adolescence. Most studies primarily focused on performance and body composition outcomes, whereas relatively few evaluated molecular and cellular mediators. Consequently, evidence concerning immunometabolic regulation, mTOR–AMPK–PGC-1α signaling, mitochondrial adaptations, muscle–bone–brain crosstalk, neuroplasticity, and biomarker responses remains limited. Accordingly, mechanistic pathways discussed elsewhere in this review should not be interpreted as directly demonstrated mechanisms in adolescent humans unless supported by adolescent-specific experimental evidence. Finally, the absence of integrated systems biology approaches, multi-omics analyses, and longitudinal study designs restricts our understanding of the complex interactions among exercise, nutrition, growth, and maturation. These methodological limitations should be considered when extrapolating current findings and highlight the need for more rigorous, mechanistically oriented, and developmentally informed research in adolescent populations.

A major limitation of the current evidence base is the limited availability of adolescent-specific mechanistic studies. Several pathways discussed in this review, including the PGC-1α/FNDC5/irisin/BDNF axis, lactate–SIRT1 signaling, and irisin-mediated bone responses, are supported substantially by animal models, cell-based experiments, or adult human studies rather than by direct investigations in adolescents. These studies are valuable for establishing biological plausibility and identifying potential molecular pathways through which exercise and nutritional factors may influence muscle, bone, and brain function. However, mechanistic findings from mice, cultured cells, or older adult populations cannot be assumed to occur with the same magnitude or physiological significance during adolescence. The same caution applies to proposed interactions among mTOR, AMPK, PGC-1α, redox signaling, and inter-organ communication, for which direct adolescent-specific evidence remains comparatively sparse. Accordingly, the mechanistic framework presented in this review should be interpreted as a hypothesis-informed synthesis rather than definitive evidence of pathway activation in adolescents. Future adolescent-specific studies incorporating longitudinal designs and direct measurements of relevant molecular, metabolic, and neurophysiological biomarkers are needed to determine whether these proposed mechanisms are operative during human development.

Practical dosing considerations are summarized in Table 8. For adolescent athletes, supplementation should be individualized according to body mass, biological maturation, training demands, habitual dietary intake, and overall energy availability. Importantly, the ranges presented should be regarded as evidence-informed practical considerations rather than universal prescriptions, because adolescent-specific data remain substantially more limited than those available in adults. Whey protein should primarily be used to help achieve adequate total dietary protein intake, whereas creatine protocols should avoid unnecessary high-dose or prolonged loading in view of the limited long-term safety evidence in adolescents. Neither supplement should be considered a substitute for adequate energy availability, dietary diversity, or a well-structured training and recovery program.

## 11 Practical applications for adolescent athletes

The findings synthesized in this narrative review underscore several important practical implications for adolescent athletes engaged in diverse sporting disciplines. Given the dynamic physiological, neuromuscular, and neurodevelopmental changes that characterize adolescence, exercise and nutritional interventions should be individualized according to biological maturation status, training demands, habitual dietary intake, and sport-specific goals. Aerobic exercise remains a fundamental component of youth athletic development due to its capacity to improve cardiovascular fitness and support metabolic and mitochondrial adaptations. However, the available evidence does not establish that the addition of whey protein or creatine is necessary for these exercise-related adaptations in adequately nourished adolescents. Regular aerobic training may also contribute to neurocognitive function and psychological wellbeing, although the extent to which these effects are mediated by specific neurotrophic pathways such as BDNF in adolescent athletes remains incompletely characterized.

From a nutritional perspective, whey protein supplementation may be strategically incorporated when dietary protein intake is insufficient or when practical circumstances make adequate protein consumption from whole foods difficult. The provision of high-quality essential amino acids, particularly leucine, provides a plausible biological basis for supporting muscle protein remodeling following exercise; however, the available adolescent trials do not establish that whey protein consistently produces superior muscle growth or athletic performance compared with adequate protein intake from the habitual diet. Whey protein may therefore be most appropriately viewed as a convenient source of high-quality protein rather than as a universal ergogenic aid, particularly for adolescent athletes exposed to high training volumes, increased recovery demands, or inadequate dietary protein intake.

Creatine supplementation may be particularly relevant to sports characterized by repeated high-intensity efforts, explosive actions, and rapid changes of direction, including soccer, basketball, wrestling, and sprint swimming. Adolescent-specific trials suggest potential improvements in selected outcomes such as repeated high-intensity performance, muscular power, jumping ability, and sport-specific performance; however, responses are not uniform across all performance measures, and evidence for endurance-dominant activities remains less consistent. Potential effects of creatine on redox regulation, mitochondrial function, and brain energy metabolism remain biologically plausible but should not currently be presented as established practical benefits in adolescent athletes because direct adolescent-specific evidence is limited.

Although exercise, protein intake, and creatine supplementation may influence overlapping metabolic and neuromuscular processes, there is currently insufficient adolescent-specific evidence to conclude that their combined use produces synergistic effects on mTOR–AMPK–PGC-1α signaling, myokine secretion, muscle–bone–brain communication, or long-term athletic development. Accordingly, these mechanisms should be regarded as plausible biological frameworks rather than established clinical outcomes.

Practical supplementation strategies should prioritize evidence-informed and individualized approaches. For whey protein, the primary objective should be to achieve an appropriate total daily protein intake, with supplementation used as a practical means of meeting this requirement when needed rather than as an obligatory post-exercise intervention. Whey protein may be consumed after exercise or incorporated into meals according to individual dietary patterns and training schedules; there is insufficient adolescent-specific evidence to conclude that a single post-exercise timing strategy is universally superior. Creatine supplementation may be administered using either a loading strategy or a lower daily dose in adult populations, but adolescent-specific evidence supporting the necessity or superiority of loading protocols is limited. Given the limited long-term safety data in adolescents, unnecessary high-dose or prolonged loading should therefore be avoided.

Nevertheless, supplementation practices in adolescents must always complement, rather than replace, a balanced and nutrient-dense diet. Adequate total energy availability, carbohydrate intake, dietary fat, micronutrient intake, hydration, sleep, and recovery should be addressed before supplementation is considered. Careful attention should also be given to safety, ethical considerations, supplement quality, and athlete education. The use of ergogenic aids in youth sport should occur only when there is a clear nutritional or performance rationale and preferably with the involvement of qualified healthcare professionals or sports dietitians familiar with adolescent athletes, particularly when medical, nutritional, or developmental considerations are present.

Creatine and whey protein supplementation in adolescents should therefore be considered within the broader context of normal growth, adequate nutrition, and individualized training needs. These supplements should not be used as substitutes for a balanced diet, and their use should be guided by a qualified healthcare or sports nutrition professional, particularly in young athletes with specific medical, nutritional, or training considerations. Before initiating supplementation, a nutritional assessment should first determine whether an actual dietary need exists; clinical assessment may be appropriate when relevant medical or safety concerns are present. Parents or guardians and adolescent athletes should also receive clear information regarding the evidence-supported purpose of supplementation, appropriate use, product quality, and potential uncertainties and risks. This precautionary approach is particularly important during adolescence because nutritional requirements, growth, biological maturation, and physiological development are still evolving. Overall, the practical priority should remain an adequately planned diet and training program, with supplementation considered as an adjunct rather than a central component of adolescent athletic development.

## 12 Future research directions

Future investigations should prioritize addressing the substantial gaps in current evidence through adequately powered, developmentally informed, and preferably longitudinal randomized controlled trials examining the effects of aerobic exercise, whey protein, and creatine supplementation individually and, where scientifically justified, in combination on growth, maturation, athletic performance, recovery, and health outcomes in adolescent populations. Future trials should address the short intervention periods, small sample sizes, and substantial heterogeneity that characterize much of the current literature, while incorporating standardized reporting of supplementation dose, adherence, habitual dietary intake, energy availability, training load, biological maturation, and adverse events. Long-term follow-up is particularly important for determining whether observed changes in performance or physiological biomarkers are sustained and whether supplementation is associated with any clinically relevant developmental consequences.

Particular emphasis should be placed on determining rather than presuming the molecular and cellular mechanisms through which exercise and nutritional interventions may interact during adolescence. Adolescent-specific mechanistic studies should directly assess pathways proposed in the present review, including mTOR–AMPK–PGC-1α signaling, mitochondrial adaptations, muscle protein turnover, phosphocreatine metabolism, and redox-sensitive pathways, rather than relying predominantly on evidence derived from animal models, cell-based experiments, or adult populations. This distinction is essential because the magnitude and physiological relevance of these pathways may differ according to pubertal stage, sex, training status, and developmental context.

Emerging concepts, including immunometabolism and muscle–bone–brain crosstalk, warrant further investigation. Future studies should determine whether changes in circulating myokines, osteokines, adipokines, inflammatory mediators, and neurotrophic factors are consistently associated with functional adaptations in adolescents and whether these biomarkers provide mechanistic information beyond conventional performance measures. Future research could investigate how exercise and nutritional interventions influence communication among skeletal muscle, bone, adipose tissue, liver, and the central nervous system through mediators such as irisin, IL-6, osteocalcin, IGF-1, BDNF, and inflammatory cytokines. However, associations between circulating biomarkers and functional outcomes should not automatically be interpreted as evidence of causal muscle–bone–brain signaling. Experimental designs incorporating repeated biomarker measurements, tissue-specific measures where ethically feasible, and appropriate temporal analyses may help clarify these relationships.

Additionally, future studies should incorporate multidimensional outcome measures extending beyond traditional performance indices to include validated measures of motor competence, cognitive function, psychological wellbeing, recovery, musculoskeletal development, and health-related quality of life. Importantly, physical literacy should be assessed directly with validated instruments when it is proposed as an outcome, because improvements in sprinting, jumping, aerobic capacity, attention, or sport-specific skills should not be considered direct evidence of improved physical literacy. Similarly, claims regarding motor learning should ideally be supported by longitudinal assessments of skill acquisition and retention rather than inferred solely from acute changes in attention or physical performance.

Given the substantial inter-individual variability observed in training and supplementation responses, future research should examine whether biological maturation, sex, baseline nutritional status, training status, energy availability, habitual diet, sleep, and other lifestyle factors explain clinically meaningful differences in responses to supplementation and exercise. Precision-nutrition approaches may ultimately be valuable, but their application in adolescent sport should be considered an emerging research direction rather than an established framework, particularly because evidence linking genetic background or gut microbiota composition to supplementation responses in adolescent athletes remains limited.

Finally, systems biology and multi-omics technologies, including genomics, transcriptomics, proteomics, and metabolomics, may help characterize the complex biological responses to exercise and nutritional interventions. Advanced computational modeling could further assist in integrating longitudinal physiological, metabolic, immunological, and neurocognitive data. Nevertheless, these approaches should complement rather than replace well-controlled clinical and functional outcomes, and their interpretation should account for developmental changes that occur independently of the intervention. Importantly, future studies should incorporate standardized safety monitoring, including renal and hepatic biomarkers where appropriate, growth velocity, pubertal maturation, bone development, adverse events, dietary intake, and supplementation adherence, particularly for longer-term creatine and protein interventions.

Collectively, these research directions would help determine which effects of exercise, whey protein, and creatine are reproducible, clinically meaningful, and specific to adolescence, while distinguishing established findings from mechanistically plausible but unconfirmed pathways. Such evidence will be necessary to develop appropriately cautious and developmentally informed nutritional and exercise recommendations for youth sport.

## 13 Conclusion

The evidence synthesized in this narrative review indicates that aerobic exercise represents an important foundation for adolescent physical and athletic development, whereas whey protein and creatine supplementation may provide context-dependent nutritional support when appropriately indicated. Current adolescent-specific evidence does not yet establish that the combined use of these interventions produces synergistic effects, and their potential interactions should therefore be regarded as an important area for future investigation rather than an established outcome.

Collectively, these interventions may influence overlapping physiological processes involved in growth, recovery, energy metabolism, and neuromuscular function, although the magnitude and clinical relevance of these effects vary considerably among individuals and sporting contexts. Aerobic exercise can support cardiovascular fitness, mitochondrial adaptations, metabolic flexibility, and oxidative capacity, with AMPK- and PGC-1α-related pathways providing plausible molecular mechanisms for some of these adaptations. Concurrently, whey protein supplementation provides essential amino acids that can support adequate protein intake and muscle protein remodeling, with leucine-sensitive mTORC1 signaling providing a plausible mechanistic basis for its anabolic effects. However, adolescent clinical trials do not consistently demonstrate superior improvements in strength, body composition, or athletic performance when whey protein is added to an already adequate diet.

Creatine supplementation has shown the most consistent adolescent-specific performance benefits in selected activities characterized by repeated high-intensity or explosive efforts, potentially through increased phosphocreatine availability and more rapid ATP resynthesis. Nevertheless, effects are not uniform across all performance domains, and evidence for endurance-dominant activities remains less consistent. Potential effects of creatine on cellular hydration, redox regulation, mitochondrial function, and brain energy metabolism remain biologically plausible but are not sufficiently established in adolescents to be considered primary practical outcomes.

Importantly, exercise and nutritional status may influence broader immunometabolic and inter-organ signaling processes involving skeletal muscle, bone, adipose tissue, and the nervous system. However, much of the proposed muscle–bone–brain crosstalk, including pathways involving myokines, osteokines, neurotrophins, and inflammatory mediators, remains supported by mechanistic, animal, cell-based, or adult evidence rather than direct adolescent-specific studies. These mechanisms should therefore be interpreted as biologically plausible frameworks rather than definitive explanations for the functional effects observed in adolescent athletes.

The available evidence supports exercise as the primary component of adolescent athletic development, with protein and creatine supplementation potentially serving as adjunctive strategies when nutritional or training circumstances justify their use. Nevertheless, the available literature remains heterogeneous, with inconsistencies attributable to variations in study duration, supplementation protocols, training modalities, participant characteristics, biological maturation, habitual dietary intake, sex distribution, and outcome measures. Moreover, evidence regarding long-term safety, dose–response relationships, developmental stage-specific responses, and sustained functional outcomes remains limited.

Overall, the current evidence supports a cautious, individualized, and food-first approach to the use of whey protein and creatine in adolescent athletes. Aerobic exercise should remain a central component of training, while supplementation should be considered as an adjunct rather than a prerequisite for adaptation or performance improvement. Whey protein may be useful when it facilitates adequate total protein intake, whereas creatine may be most relevant to selected sports involving repeated high-intensity efforts. Decisions regarding supplementation should consider biological maturation, total dietary intake, energy availability, training demands, sport-specific goals, medical history, hydration, and product quality. However, additional long-term, adequately powered, adolescent-specific, and mechanistically informed investigations are required to determine the reproducibility and clinical significance of these effects, establish appropriate dose–response relationships, clarify developmental and sex-specific responses, and determine whether the proposed molecular and inter-organ mechanisms are directly operative during adolescence. Such evidence will be essential for distinguishing established benefits from plausible but unconfirmed mechanisms and for developing safe, developmentally informed, and evidence-based recommendations for adolescent athletes.
